# The Condition of Air Pollution in Kraków, Poland, in 2005–2020, with Health Risk Assessment

**DOI:** 10.3390/ijerph17176063

**Published:** 2020-08-20

**Authors:** Paulina Traczyk, Agnieszka Gruszecka-Kosowska

**Affiliations:** Department of Environmental Protection, Geophysics and Environmental Protection, Faculty of Geology, AGH University of Science and Technology, Al. Mickiewicza 30, 30-059 Krakow, Poland; p.traczyk94@gmail.com

**Keywords:** air pollution, air quality index, particulate matter, enrichment factors, health risk assessment, COVID-19

## Abstract

Aims: Air quality changes with human health risk assessment were investigated. Methods: The measurement results obtained by the Regional Environmental Protection Inspectorate (REPI) in Kraków and our deposited particulate-matter (PM) analysis, as well as United States Environmental Protection Agency (USEPA) methodology of risk assessment were used in the study. Results: Annual pollutant contents kept decreasing, with the exception of O_3_. However, the permissible annual levels were exceeded in the cases of PM10, PM2.5, and NO_2_. Increased contents of SO_2_, CO, C_6_H_6_, PM10, and PM2.5, as well as of As, Pb, Cd, Ni, and polycyclic aromatic hydrocarbons (PAHs) in PM particles during winter months indicated that house heating was the source of pollution. Due to no significant change in the monthly NO_2_ contents, this measurement was used as an indicator of traffic sources of pollution. In winter months, the allowable 24 h PM2.5 and PM10 contents were constantly exceeded. PM was identified as the most significant air pollutant. Enrichment factors revealed that deposited PM was enriched with heavy metals. The potential ecological risk (ERI) was determined to be very high for Cd, considerable for Zn, and low for As, Co, Cr, Cu, Ni, Pb, and Tl. The total non-carcinogenic risk indices (HQ) for both adults (HQ = 15.0) and children (HQ = 26.4) exceeded the acceptable value of 1. The total carcinogenic risk indices (CR) for both adults (CR = 1.51 × 10^−4^) and children (CR = 1.77 × 10^−4^) exceeded the acceptable level of 1 × 10^−4^. Conclusions: In the years 2005–2020, a general decreasing tendency of annual pollutant contents was observed. However, the permissible contaminant contents were still exceeded. PM2.5, BaP, PM10, and NO_2_ were determined as the most dangerous pollutants in inhalational pathway.

## 1. Introduction

**Figure 1:**
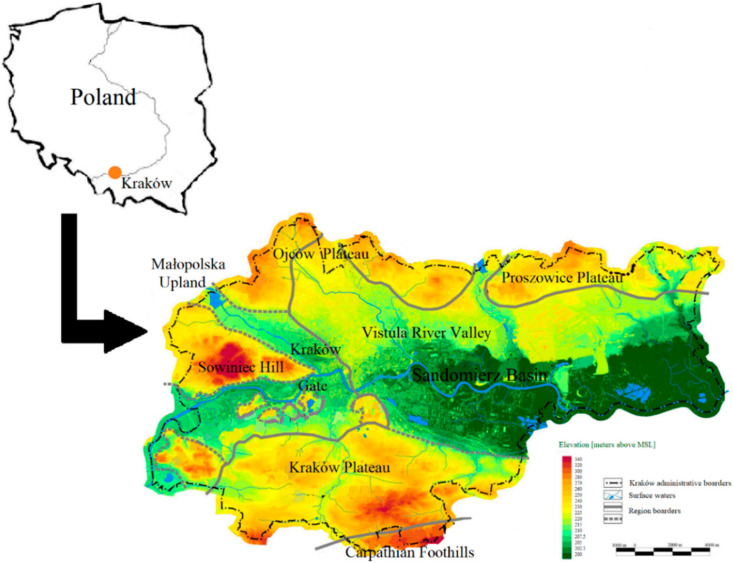
Geographic location of Kraków in Poland and elevation changes of the city (modified after [19]).

For several years, Kraków has been said to represent an example of the cities with poor air quality. Kraków has suffered high concentrations of gaseous and dust pollutants in air since the 1950s, and that was associated with the development of industry and operation of combined heat and power plants and steelworks in Kraków [1]. However, since the 1990s, a decrease of air pollution has been observed, owing to the implementation of control technologies with lower emission standards and decentralisation of the government, as well as privatisation processes [1,2,3]. Nevertheless, the World Health Organisation (WHO) placed Kraków at position no. 11 on the list of the 50 most polluted cities of the European Union [4]. Later, according to the 2018 update, Kraków was moved to position no. 8 on that list [5]. The current air pollution condition in Kraków results from the operation of power plants, low emissions [6,7], and traffic [8]. Moreover, the city is surrounded by communes, where poor-quality coal stoves are still the dominant house heating systems. Consequently, the inflow of emissions from neighbouring towns causes a deterioration of air quality in Kraków. Despite the fact that a total ban on burning coal, wood, and other solid fuels in boiler houses, stoves, and fireplaces was imposed in the city of Kraków on 1 September 2019 [9], air pollution continues to be excessive. The condition of air in Kraków was widely investigated under several research projects [10,11,12,13,14,15,16,17,18]. Moreover, air quality studies in this particular city are not easy due to its geographical and topographical condition. Kraków is located in southern part of Poland and borders the Carpathian Foothills in the south, Silesian-Kraków Upland in the west, Kraków Upland in the north, and Sandomierz Basin in the east (Figure 1) [19]. In addition, within the administrative boundaries of Kraków, uplands are observed in northern, western, and southern parts of the city, while lowlands are observed in eastern Kraków. The maximum height amplitude in the investigated area is approximately 140 m above mean sea level (MSL) between the Vistula River Valley in the east and Sowiniec Hill in the west [19]. The location of Kraków in the Vistula River valley, dividing the city into the northern and southern parts, determines the shape of the observed wind rose [20] (Figure S1). West winds are dominating, and east ones occur with high frequency [20]. Land relief in a concave form defines the latitudinal direction of city ventilation [20] (Figure S2). Existing urban and industrial buildings reduce the speed of winds and modify the wind directions [20].

Air pollution is nowadays considered to be one of the most important factors affecting human health. Poor air quality causes damages in the respiratory tract and cardiovascular systems [21,22,23,24,25], as well as an increase of premature death rates in populations [26,27,28,29,30]. Specific attention was lately paid to particulate matter (PM). PM particles can adsorb other harmful substances on their surfaces [31], and consequently, they can cause additional adverse health effects after entering human bodies [32]. Street dust was described as the most effective and sensitive indicator of urban environment pollution, especially by heavy metals [33]. The enrichment of dust with heavy metals can be caused by anthropogenic sources, including means of transportation, industrial plants, fossil fuel burning, or construction works [34,35].

In recent years, the residents of Kraków have been trying to resolve effectively the poor air quality problem. The movement was started in 2012 by the implementation of several social campaigns undertaken by the Kraków Smog Alert, a non-government organisation [3]. However, evident changes required not only considerable expenditures, but also time measured in years. Currently, the residents and visitors are affected by adverse health effects due to poor air quality inhaled. According to the authors’ best knowledge, the first attempts at calculating human health risk among the Kraków inhabitants were described in research conducted by Samek [16], Gruszecka and Wdowin [36], Pachurka et al. [37], and Gruszecka-Kosowska [38].

Taking the above into consideration, the objectives of our present study were selected as follows: (1) to determine the changes occurring in general air quality in Kraków in the last 15 years (2005–2020), (2) to identify the most significant air pollutants, from the viewpoint of health, and (3) to assess human health risk for the Kraków inhabitants, arising from the exposure to the ambient-air contaminants.

## 2. Materials and Methods

### 2.1. Concentration of Pollutants in the Ambient Air in Kraków

To perform long-term and short-term air-quality change analyses, the authors investigated the results of the air monitoring measurements obtained by the Regional Environmental Protection Inspectorate (REPI) in Kraków (2005–2020) collected from up to nine monitoring stations located in the city (Table S1). The measurement results have been published in the Internet since 2007 [39], while those concerning pollutant concentrations in 2005 and 2006 were taken from the REPI reports [40,41,42,43,44,45].

The following pollutants measured by the REPI were investigated in the present study: benzene (C_6_H_6_), carbon oxide (CO), nitrogen dioxide (NO_2_), sulphur dioxide (SO_2_), and particulate matters PM10 and PM2.5, as well as the following pollutants measured in PM10 particles (PM10): arsenic (As), lead (Pb), cadmium (Cd), nickel (Ni), benzo[a]anthracene (BaA), benzo[a]pyrene (BaP), benzo[b]fluoranthene (BbF), benzo[j]fluoranthene (BjF), benzo[k]fluoranthene (BkF), and dibenzo[a,h]anthracene (DBahA).

### 2.2. Air Quality Standards

To determine the air quality in Kraków, the REPI measurement results were compared to the Polish permissible levels of pollutants determined in the Regulation of the Minister of the Environment concerning the levels of certain substances in the air [46]. Based on that legal act, the permissible annual concentrations of air contaminants were determined as follows: C_6_H_6_: 5 µg/m^3^, NO_2_: 40 µg/m^3^, SO_2_: 125 µg/m^3^, CO: 10,000 µg/m^3^, PM10: 40 µg/m^3^, PM2.5: 25 µg/m^3^ (20 µg/m^3^ since 1 January 2020), and Pb (PM10): 0.5 µg/m^3^. The Polish regulation also determined 24 h (24 h) permissible levels for the following contaminants: SO_2_ (125 µg/m^3^) and PM10 (50 µg/m^3^). Since the Polish regulation did not specify any permissible 24 h PM2.5 content, that value was adopted from the World Health Organisation air quality guidelines and was set at 25 µg/m^3^ [47]. One hour (1 h) permissible contaminant levels were also defined in the Polish regulation for NO_2_ and SO_2_ and were equal to 200 µg/m^3^ and 350 µg/m^3^, respectively, as well as an eight-hour (8 h) permissible level for O_3_ equal to 120 µg/m^3^. For other heavy metals in PM10, also the European Union and the United Kingdom recommended values were used [48]. The target values were set as follows: As (PM10): 6 ng/m^3^, Cd (PM10): 5 ng/m^3^, and Ni (PM10): 20 ng/m^3^.

### 2.3. Polish Air Quality Index

The air quality index (AQI) is a widely recognised index that is easily understood by the public, due to the presentation of air contamination on a numerical scale, using a colour-intensity coding. In our study, 1 h air quality measurements were used for a better visualisation of the Polish AQI index values. As to individual pollutants, the air quality index is defined by the ranges of 1 h concentrations of pollutants (Table S2) [39]. The Polish AQI index also reflects negative health effects, in direct proportion to the AQI scale growth (Table S3) [39].

### 2.4. Enrichment Factors of PM with PHEs

Particulate matter (PM) becomes a crucial contaminant since it contains other pollutants adsorbed on particle surfaces, and special attention is paid to heavy metals in that respect. Thus, in our study, the enrichment factors of particulate matter, with potentially harmful elements (PHEs), were calculated. Since the Polish Regional Environmental Protection Inspectorate (REPI) measured only four heavy metals (As, Cd, Ni, and Pb) in PM particles, our analysis also concerned selected concentrations of PHEs, identified in deposited PM particles by Gruszecka-Kosowska and Wdowin [36]. As to the enrichment factors, our specific calculations refer to the PHE contents in deposited particulate matter, as presented in Gruszecka-Kosowska [49].

The following enrichment factors of PM with PHEs were investigated: geoaccumulation index (I_geo_), contamination factor (CF), enrichment factor (EF), ecological risk index (ERI), and modified hazard quotient (mHQ). The I_geo_ and CF factors determine the accumulation of elements in relation to background values. In our study, local geochemical values were taken from Kabata-Pendias [50]. The EF factor describes element enrichment, in respect of the elements with a low variability of occurrence, and Fe was chosen for that purpose in our study. To calculate the EF values, both concentrations from upper continental crust [51] and local geochemical values [50] were taken. The ERI and mHQ indices determine ecological risk by comparing metal concentrations to the synoptic adverse ecological effect distributions, in respect of slightly differing threshold levels [52]. Detailed descriptions of enrichment indices applied in the present study are given in Table S4, as presented in Gruszecka-Kosowska [49].

### 2.5. Health Risk Assessment

Health risk was assessed based on the United States Environmental Protection Agency methodology [53]. In our research, the resident scenario was analysed for adults and children (0–6 years). The following exposure pathways for the residents of Kraków were investigated in our research: inhalation of the contaminants, being present in the ambient air, inhalation of potentially toxic elements (PHEs), being present in deposited particulate matter (PM) particles, accidental ingestion of PHEs, being present in deposited PM particles, and dermal contact with PHEs, being present in deposited PM particles. The contents of pollutants in the ambient air were obtained from the data collected by the Regional Environmental Protection Inspectorate (REPI) in Kraków [39] as mean values, upon averaging the measurement results of the period from 2005 to 2020, as well as from the data originating from up to nine monitoring stations located in the city. The PHE contents in deposited PM were taken from research conducted by Gruszecka-Kosowska and Wdowin [36] on PHEs in deposited PM, as mean values of the investigated elements, as described in Gruszecka-Kosowska [49].

To estimate the daily intake of contaminates via the inhalation route, based on the REPI measurement exposure concentration (EC_inh air_), the values were calculated according to Equation (1) [54], while the average daily dose (ADD_inh air_) values were calculated according to Equation (2) [53], depending on the availability of the toxicological data required for risk value calculations:

EC_inh air_ = (C_air_ × ET × EF × ED)/AT (1)

ADD_inh air_ = (C_air_ × IR_inh_ × EF × ED)/(BW × AT). (2)

For estimation of the daily intake of the PHEs being present in redeposited PM particles via the inhalation route, the exposure concentration (EC_inh PM_) values were calculated based on Equation (3) [55], while the average daily dose (ADD_inh PM_) values were calculated according to Equation (4) [56], depending on the availability of the toxicological data needed for risk values calculations:

EC_inh PM_ = (C_PM_ × ET × EF × ED)/(PEF × AT) (3)

ADD_inh PM_ = (C_PM_ × IR_inh_ × AF × EF × ED)/(PEF × BW × AT). (4)

To estimate the daily intake of PHEs being present in redeposited PM particles via the ingestion route, the average daily dose (ADD_ing PM_) values were calculated according to Equation (5) [55]:

ADD_ing PM_ = (C_PM_ × CF × IR_ing_ × FI × EF × ED × RBA)/(BW × AT). (5)

To estimate the daily intake of PHEs being present in redeposited PM particles via the dermal contact route, the average daily dose (ADD_der PM_) values were calculated according to Equation (6) [55]:

ADD_der PM_ = (C_PM_ × CF × AF × ABS_d_ × EF × ED × EV × SA)/(BW × AT), (6)

where EC, exposure concentration (mg/m^3^); ADD, average daily dose (mg/kg-day); C_air_, contaminant concentration in air (measured values were converted to mg/m^3^); C_PM_, concentration of each element in deposited PM (mg/kg); IR, intake rate (m^3^/day or mg/kg); PEF, particle emission factor (m^3^/kg); CF, unit conversion factor (10^−6^ kg/mg); FI, fraction ingested from contaminated source (unitless); RBA, relative bioavailability factor (unitless); AF, adherence factor of PM to skin (mg/cm^2^ event); ABS_d_, dermal absorption factor (unitless); EV, event frequency (events/day); SA, skin surface area available for contact (cm^2^); ET, exposure time (h/day or days/year); EF, exposure frequency (days/year); ED, exposure duration (years; BW, body weight (kg); AT, averaging time (ED in years × 365 days/year × 24 h/day in hours or ED in years × 365 days/year in days; for non-carcinogens ED = 24 years, for carcinogens ED = 70 years [54]).

The exposure parameters used for the risk assessment calculations under the resident scenario are given in Table S5.

To determine the non-carcinogenic and carcinogenic risks for the residents of Kraków in four investigated exposure pathways, the HQ and CR values were calculated, respectively. The values of hazard indices (HQ) were calculated using Equations (7) and Equations (8) [55]. The values of carcinogenic risk (CR) were calculated using Equations (9) and Equations (10) [55].

HQ = EC/RfC,(7)

HQ = ADD/RfD,(8)

CR = EC × IUR,(9)

CR = ADD × SF,(10)

where HQ, hazard quotient (unitless); CR, carcinogenic risk (unitless); ADD, average daily dose (mg/kg-day), EC, exposure concentration (mg/m^3^), RfC, reference concentration (mg/m^3^); RfD, reference dose (mg/kg-day); IUR, inhalation unit risk (mg/m^3^); SF, slope factor (mg/kg-day)^−1^.

The values of toxicological parameters, used for the calculations of the resident scenario risk assessment, are given in Table S6.

The target risk value was set to be equal to 1 (HQ = 1) for non-carcinogenic risk, for both the individual contaminants being investigated (individual HQ values) and total non-carcinogenic risk, which was defined as the sum of all the calculated HQ values. As for carcinogenic risk, the acceptable risk level was set to be equal to 1 × 10^−6^ for an individual contaminant and equal to 1 × 10^−4^ for the sum of carcinogenic contaminants [53,57].

## 3. Results

### 3.1. Pollutant Content and Air Quality Changes in 2005–2020

Upon our analysis of the mean annual investigated pollutant concentrations collected from all the monitoring stations, in the period of the last 15 years (Table S7), we observed a general tendency of content decrease, with the exception of O_3_, whose increased levels were identified. Despite the above-described general tendency, permissible annual contents of the investigated pollutants (if determined) were exceeded in all the investigated years in the cases of PM10 and PM2.5 (except for 2019) and in the case of NO_2_ (except for 2014 and 2019). However, excessive C_6_H_6_ levels of annual permissible rates were determined in 2005 and 2006 (Table S7). For our further investigations, 2018 was chosen as the most recent year, with a significant excess of permissible levels of pollutants. The investigated pollutants contents, which were calculated as the mean monthly values of the data originating from all the monitoring stations in Kraków, are presented in Table S8. Higher contents of SO_2_, CO, C_6_H_6_, PM10, PM2.5, heavy metals, and polycyclic aromatic hydrocarbons (PAHs) were observed in winter months in comparison to summer months. In addition, 24 h air pollutant contents, calculated as mean values, collected from all the monitoring stations in Kraków, were investigated. March 2018 and July 2018 were chosen as model winter and summer months, respectively to show the most spectacular (the highest) daily values of investigated pollutants. Upon our analysis of 24 h selected pollutant contents in March 2018 (Table S9), it was observed that the PM2.5 contents exceeded the WHO recommended value of 25 µg/m^3^ in all days; as to PM10, the same happened in the first 11 and last nine days of March. Our analysis of 24 h selected pollutant contents in July 2018 (Table S10) was concluded with the observation that in the summer months, an excess of permissible level of 120 µg/m^3^ was observed only in the case of O_3_.

We applied the Polish AQI index to present hourly air-pollutant rate changes, with correlated health information. March 2018 was chosen for our analysis as the month with generally significant pollutant contents. The worst situation was observed in the cases of PM2.5 (Table 1) and PM10 (Table 2), as well as C_6_H_6_ (Table 3). In March, the Polish AQI index was determined to range from average to very bad for PM2.5 and PM10, and from moderate to average for C_6_H_6_. The general air quality index for NO_2_ (Table 4) was estimated to be very good to good, with several hours of moderate air quality. As to NO_2_, the increase of its contents was observed in the morning and evening hours. O_3_ displayed increased contents during the whole day (Table 5), from 18 to 19 March 2018. In the cases of SO_2_ (Table S11) and CO (Table S12), a very good air quality index was determined for the whole month of March 2018, based on mean 24 h concentrations.

The decreases in the monthly contents recorded in the first five months of 2020 (Table S13) were observed for PM2.5, PM10, and C_6_H_6_, and slight decreases of monthly contents were also observed in the case of NO_2_.

### 3.2. PM Enrichment Factors

Based on the above conclusions, particulate matter was determined to be the most significant contaminant. Since PM particles adsorb various pollutants, our subsequent investigations focussed on heavy metal contents in deposited PM particles. The calculated values of the applied enrichment indices and of the corresponding classes are presented in Table 6. Enrichment factors calculated for average heavy metal content data and obtained from the Regional Environmental Protection Inspectorate (REPI) measurements revealed that suspended PM10, according to I_geo_ classification, remained practically uncontaminated (class 0) with Cd and Pb, moderately to heavily contaminated (class 3) with As, and extremely contaminated (class 6) with Ni. The CF index indicated a low contamination of PM10 with As, Ni, and Pb, although a very high contamination with Cd was identified. The EF values (calculated here as the mean values of EF established for various background values) indicated a moderately severe enrichment of PM10 with Cd, while in the cases of As, Ni, and Pb, no enrichment was found. The calculated values of ERI indicated high ecological risk in respect of Cd and low ecological risk in respect of As, Ni, and Pb. Ecological risk, defined on the basis of the mHQ index, revealed a very low severity of contamination with Cd and none to very low severity of contamination with As, Ni, and Pb.

The results of the calculations of heavy metal contents in deposited PM were described in a conference paper by Gruszecka-Kosowska [49]. Based on the calculated I_geo_ values in deposited PM samples, heavy metal accumulation was found to be the highest for As, Ba, Cr, Cu, Li, Mn, Ni, Pb, Sr, Ti, V, and Zn (class 6), as well as Co and Sn (class 5). Instead, in the cases of Be, Cd, and Tl, the calculated I_geo_ values indicated no accumulation (class 0). On the other hand, the calculated CF values revealed a very high contamination of deposited PM samples with Cd and Zn, considerable contamination with As, Pb, and Sn, and moderate contamination with Cu and Li. As to the remaining investigated elements, the CF values indicated low contamination. Besides, the EF values indicated that deposited PM samples were extremely severely enriched with Zn, moderately to severely enriched with Sn, and severely enriched with Cd. A minor enrichment of PM with Cu was observed. As to the remaining investigated elements, the EF values indicated no enrichment. The calculated values of ERI indicated a very high ecological risk of deposited PM samples only in the case of Cd and considerable ecological risk in respect of Zn. Low ecological risk was determined in respect of As, Co, Cr, Cu, Ni, Pb, and Tl. Ecological risk, based on the mHQ index, revealed an extreme severity of contamination of deposited PM samples with Zn and a considerable severity of contamination with Cr. As to As, Cu, and Pb, moderate severity contamination was indicated, while in the cases of Cd and Ni, low-severity contamination was indicated. The values of mHQ were not defined for Ba, Be, Co, Li, Mn, Sn, Sr, Ti, Tl, and V due to the lack of adverse ecological effect values.

### 3.3. Health Risk Assessment

Since the Polish AQI index provides general health risk information focussed on resident actions from “stay at home” to “safe intense physical activity”, our risk assessment was calculated to define the reliable risk for inhabitants [58]. Health risk assessment of the investigated air pollutants was calculated on such a basis that if the RfC values for non-carcinogenic and the IUR values for carcinogenic pollutants were available, the relevant equations were used in the first place.

The calculated estimated daily intakes are presented in Table S14. For our calculations, the pollutant content mean values from the available monitoring stations and the last 15 years were used. The health risk assessment values for the residents of Kraków are presented in Table 7. The total non-carcinogenic risk, calculated as the sum of single non-carcinogenic pollutant rates, exceeded the acceptable level significantly. The risk rate was equal to 15.0 in adult residents and 26.4 in children. The total carcinogenic risk, which was calculated as the sum of single carcinogenic pollutant rates, exceeded the acceptable level as well. The risk rate was equal to 1.51 × 10^−4^ in adult residents and 1.77 × 10^−4^ in children.

Regarding the inhalation of deposited PM, the decreasing order of the largest impact on non-carcinogenic risk values, in both adults and children, was determined as follows: Mn > Al > As > Cr(VI) > Co > V > Ni > Ba > Cd > Be > Fe > Zr > Pb > Zn > Li > Cu > Sr > Cr(III) > Sn, and, as regards carcinogenic risk: Pb > Cr(VI) > Ni > As > Co > Cd > Be. Regarding the accidental ingestion of deposited PM, the decreasing order of the largest impact on non-carcinogenic was determined as follows: in adults, Fe > Zr > As > Cr(VI) > Mn > Pb > Zn > Li > Co > Al > V > Cu > Cd > Ni > Ba > Be > Sr > Cr(III) > Sn; in children, Fe > As > Zr > Cr(VI) > Mn > Pb > Zn > Al > Li > Co > V > Cu > Cd > Ni > Ba > Be > Sr > Cr(III) > Sn. Regarding the accidental ingestion of deposited PM, the decreasing order of the largest impact on carcinogenic risk, in both adults and children, was determined as follows: Cr(VI) > As > Pb. Regarding dermal contact with deposited PM, the decreasing order of the largest impact on non-carcinogenic risk, in both adults and children, was determined as follows: Fe > Zr > Cr(VI) > Mn > Pb > Zn > Li > Co > Al > V > Cu > As > Ba > Be > Sr > Cr(III) > Sn > Ni > Cd, and as regards carcinogenic risk, Cr(VI) > Pb > As.

## 4. Discussion

Our research project presented here was based on the average data obtained from all the monitoring stations installed in Kraków. The general tendency of pollutant contents decrease (except for O_3_) in the ambient air over the years included might be caused by the following: fuel desulphurisation, liquidation of heavy industry and implementation of ecological technologies in industry, gradual modernisation of car fleets in Kraków, poor-quality stove replacement, and using better-quality fuels for house heating purposes until the city of Kraków introduced the prohibition of burning coal and wood (not to mention waste) on 1 September 2019. The significant decrease in the mean year contents of pollutants between 2006 and 2007 might have been caused by cold winters in 2006 and in previous years. It might cause the increase in emissions from heating sources, which, in the absence of conditions for the spread of pollutants, resulted in an increase in their concentration in the air, i.e., PM and SO_2_ [59]. Moreover, during cold weather, inhabitants use individual vehicles more often than public transport, which might have caused the increase in the NO_2_ contents in the air. On the other hand, the factors affecting the increase in the O_3_ contents might involve increased air temperatures and the presence of other air contaminants, i.e., NO_2_, CO, and volatile organic compounds (VOCs). Observed higher contents of heavy metals and polycyclic aromatic hydrocarbons (PAHs) in winter months in comparison to summer months could result from their content in PM particles [60]. Air quality improvement in Kraków is hindered by the adverse location of the city in the river valley, which is described in the Introduction section. Besides, poor-quality air keeps flowing into the city from the surrounding small towns and villages where strict control measures of solid-fuel burning for house heating purposes have not been introduced. Moreover, ventilation of the city is also reduced as a result of constant development blocking local air-flow corridors. Traffic remains a continuous problem because the number of vehicles keeps increasing in Kraków, while the average age of cars was estimated at about 14 years in Poland in 2018. Higher contents of SO_2_, CO, C_6_H_6_, PM10, and PM2.5 and heavy metals and PAHs, contained in PM particles [60] observed in winter months in comparison to summer months, indicated that local emission sources were the main causes of pollution [6,7]. It needs to be added here that in the past several years, winters become milder, and freezing temperatures shifted from November to March in Poland [61,62]. On the contrary, higher O_3_ pollution contents were observed in summer rather than winter months. That again could point at summer insolation being the main source of that pollutant [63]. In the case of NO_2_, no significant changes in monthly contents were observed, which indicated that traffic was the main source of that pollutant in Kraków [64]. Besides, the residents used their vehicles more frequently in cold and rainy weather, which contributed to the increase of traffic and traffic jams. On the other hand, the slight decreases recorded in the summer months could be correlated with school and college holidays, reducing traffic in the university city of Kraków. Exceedance of 24 h contents of PM2.5 and PM10 in March 2018 could be correlated with cold weather during the first 11 and last nine days of the month and low wind speed generally below 5.4 m/s representing up to a gentle breeze in the Beaufort scale of wind speed, indicating that house heating was the main cause of pollution. No correlation with weekdays or weekends was observed in that case. On the contrary, an exceedance of 24 h contents of O_3_ in July 2018 could be correlated with high temperatures occurring on 4–6 July 2018 and 21–22 July 2018 as well as with the low wind speed during these days. In addition, in that case, no correlation with weekdays or weekends was observed. According to the Polish AQI index, increased contents of PM10, PM2.5, and C_6_H_6_ in the first 11 days and last nine days of March, especially from 20:00 to 09:00 the following day, indicated that the figures were correlated with poor-quality fuel and waste burning for house heating purposes in equally poor-quality stoves. The increase of NO_2_ contents was observed in the morning and evening hours, which could indicate the effect of traffic during the rush hours. Increased contents of O_3_ during the whole day, from 18 to 19 March 2018, could point at a correlation with high temperatures and moderate wind speed during those days. Besides, during that whole period in March, high contents of pollutants in midday hours could be associated with insolation. The calculated EF values indicated anthropogenic sources of elements (EF > 30) only in the case of Zn. As for Cd, a small proportion of anthropogenic sources was determined. Regarding Pb and Sn, non-crustal sources of elements were revealed. Crustal sources of elements were defined for As, Ba, Be, Co, Cr, Cu, Li, Mn, Ni, Sr, Ti, Tl, and V.

One could expect that during the lockdown and “stay home” campaign, from March to May 2020, air quality should have significantly improved in the context of the COVID-19 pandemic crisis, while the pollutant contents should have effectively decreased, since people travelled less often. The decreases in the monthly contents observed for PM2.5, PM10, and C_6_H_6_ could be also caused by high temperatures and reduced house heating requirements. Slight decreases of monthly contents in the case of NO_2_ could have been caused by reduced traffic rates in Kraków, owing to the lockdown. Upon our analysis of the contents of the above-mentioned pollutants in March 2020 and March 2018, a double decrease in contents was observed in March 2020 in comparison to March 2018. However, that decrease could have also been caused by milder winters and the implementation of the antismog resolution in Kraków. However, daily and hourly air-pollutant content changes in the first five months of 2020 did not confirm the existence of such a trend. That could have resulted from the fact that some residents still had to work out of home and traffic remained the critical cause of air pollution in large cities. Moreover, during the lockdown in Kraków, dry weather and a lack of rainfall caused contaminants to remain suspended in the air, with the occurrence of the resuspension of contaminants from the ground. Additionally, low night-time temperatures caused an increase of fuel burning for heating purposes, as the majority of residents stayed at home during that period. On the other hand, air quality in Kraków itself, as regards the PM contents, improved in comparison to that of the surrounding communes where the antismog resolution had not been adopted. That relationship could be observed on the Airly maps, which are based on a large number of air-pollution sensor measurements in the Airly network [65]. Unfortunately, the unfavourable geographic location of Kraków prevented ventilation of the city and worsened air quality, owing to the inflow of air masses from surrounding areas.

Considering the analysed exposure pathways, the values of the decreasing participation in non-carcinogenic risk, in both adults and children, were arranged as follows: inhalation > dermal contact > accidental ingestion. As to carcinogenic risk, the decreasing participation of exposure pathways, in both adults and children, was as follows: dermal contact > ingestion > inhalation. Depending on the availability of data regarding non-carcinogenic risk, the largest impact of the ambient air on the inhalational pathway was determined as follows, in decreasing order: PM2.5 > PM10 > BaP(PM10) > NO_2_ > C_6_H_6_ > As (PM10) > Cd (PM10) > Ni (PM10) > Pb (PM10). As to the carcinogenic risk of the ambient air in the inhalational pathway, the decreasing order of the largest impact on risk values was set as follows: Pb (PM10) > C_6_H_6_ > As (PM10) > BaP (PM10) > Cd (PM10) > DBahA (PM10) > BaA (PM10) > BjF (PM10) > BbF (PM10) > BkF (PM10) > Ni (PM10).

### Limitations and Strengths of the Study

However, those data could falsify the real trends developing in particular districts of the city, as they strongly depended on the local atmospheric and topographic conditions [66,67]. Nevertheless, in our research, the average Kraków values were investigated, taking into account the long-term impact on human health. Besides, when describing the general tendency of changes in pollution contents, local atmospheric and topographic conditions should also be considered [68]. However, again, the most important conclusion, which was drawn from this point of view, was that in the past several years, the contents of some pollutants, mainly of PM, exceeded significantly and constantly either the permissible values or the recommended values when the former values were not available [48]. As our PM analysis determined, the most significant pollutant enrichment factors were found in deposited PM samples, which was confirmed by the results of Li et al. [69], Men et al. [70], and Jahandari [71]. In our approach, heavy metals and PAHs were not analysed, as they were measured in PM10, and no permissible or recommended values were available for those substances. Our analysis further concluded that enrichment factor values from the risk assessment point of view were underestimated in the suspended PM, since measured results were available only in respect of four metals and only for the PM10 fraction. Thus, according to the conservative risk assessment principle, the enrichment factor values of deposited PM were used in our health risk assessment. Moreover, hexavalent and trivalent Cr were assumed in maximum concentrations in our risk assessment at the same time, since only general chromium was determined in our laboratory analysis. The heavy metal speciation in the PM is crucial [72]; however, the calculated risk values for both types of chromium were irrelevant here, in comparison to the shares of other pollutants in that case scenario. As to Ba, Be, Li, Mn, Sn, Sr, Ti, and V, ecological risk values were not defined due to the lack of adverse ecological effect values. Additionally, since health risk assessment depends on toxicological data mostly, there is no one and only method for risk calculation. Thus, depending on the input data available and the approach applied, results may differ [36,38] and generate inaccuracies when comparing such results. Nevertheless, the main goal of our investigations was to determine the long-term health impact on the Kraków residents, because a sufficient improvement of air quality will take years.

## 5. Conclusions

Our studies revealed a general decreasing tendency of annual pollutant contents on the basis of the investigated pollutant data available for the last 15 years. Annual permissible pollutant levels were exceeded in almost all those years, in the cases of PM10, PM2.5, and NO_2_. High contents of SO_2_, CO, C_6_H_6_, PM10, and PM2.5, as well as those of As, Pb, Cd, Ni, and PAHs were observed in PM particles in winter rather than in summer months, indicating that burning solid fuels for house heating purposes was the main source of pollution. Regarding NO_2_, no significant changes were observed in monthly contents, which indicated that traffic was the main source of that pollutant in Kraków. In winter months, the recommended 24 h PM2.5 and PM10 contents were constantly exceeded. Occasionally in summer months, the excess of permissible 24 h level of O_3_ was determined, which was correlated with high temperatures and the presence of ozone precursors in the ambient air. Particulate matter was defined as the most significant air pollutant, while the calculated enrichment factors revealed a significant PM enrichment with heavy metals. Total non-carcinogenic risk values exceeded the acceptable levels, and they were equal to 15.0 in adults and 26.4 in children. Total carcinogenic risk exceeded the acceptable levels as well, since the cancer risk value was equal to 1.51 × 10^−4^ in adults and 1.77 × 10^−4^ in children. The pollutants generating the highest values of non-carcinogenic risk were PM2.5, BaP, PM10, and NO_2_ in the inhalational pathway. The highest carcinogenic risk values were generated by Pb and Cr(VI) in the inhalational pathway and Pb, As, and Cr(VI) in the accidental ingestion and dermal contact pathways. Our health risk assessment, based on the resident exposure scenario, revealed a significant health risk for the residents arising from poor air quality in Kraków.

## Figures and Tables

**Table 1:** Daily and hourly PM2.5 content changes, with the hourly Polish air quality index (AQI), average values for Kraków, March 2018 [39] (weekend marking in grey).

PM2.5																															
Day	1	2	3	4	5	6	7	8	9	10	11	12	13	14	15	16	17	18	19	20	21	22	23	24	25	26	27	28	29	30	31
Hour																															
01:00	80	73	52	184	149	118	109	77	95	93	93	37	8	58	46	52	14	18	15	33	65	62	119	105	85	56	85	24	52	64	37
02:00	87	86	54	185	156	119	100	73	101	98	96	48	5	62	51	46	11	14	15	29	63	57	123	110	87	58	84	28	52	61	33
03:00	100	83	57	182	159	130	102	74	102	93	94	37	5	57	54	44	11	16	16	32	71	52	127	115	91	58	83	23	42	56	35
04:00	109	91	60	184	146	131	95	74	90	90	92	26	5	56	48	48	12	14	16	31	81	45	122	111	98	58	81	26	47	42	32
05:00	112	93	70	179	142	122	103	74	87	93	92	20	5	53	32	45	10	13	15	31	86	41	112	110	101	67	77	26	37	42	32
06:00	111	94	78	161	124	119	100	77	98	92	89	20	8	58	29	41	10	14	15	36	90	41	103	109	97	68	72	35	34	45	29
07:00	117	90	87	160	144	106	85	79	100	93	83	19	12	59	27	38	13	14	18	40	59	37	97	104	94	70	75	40	34	46	28
08:00	144	98	88	151	154	96	93	74	90	96	86	26	19	58	24	38	17	16	18	36	18	32	90	94	91	76	83	42	37	38	28
09:00	113	117	75	137	145	95	104	63	42	101	69	31	14	44	22	37	15	15	18	31	14	27	76	89	79	68	83	42	49	27	33
10:00	68	98	69	105	146	90	115	42	31	69	51	29	11	25	19	44	16	18	21	31	14	19	51	72	54	53	56	44	32	13	25
11:00	46	89	59	70	141	73	97	29	23	45	30	27	10	24	17	40	15	16	21	31	14	17	35	63	44	38	40	37	24	11	31
12:00	50	80	57	68	102	58	59	18	19	34	20	18	8	25	18	38	17	14	22	37	14	18	40	56	43	29	33	28	15	10	30
13:00	52	77	55	54	92	57	26	14	14	22	17	10	8	28	19	39	17	15	24	38	11	15	38	34	35	23	31	28	17	11	31
14:00	61	82	55	53	73	62	24	11	13	15	13	8	8	19	18	37	16	21	24	39	11	18	37	27	30	21	34	26	19	12	23
15:00	66	75	51	53	67	57	27	10	13	15	13	7	10	16	20	38	19	24	25	37	12	16	37	18	30	20	25	29	26	10	14
16:00	68	61	47	57	59	56	35	14	12	18	12	6	11	22	19	33	16	16	25	27	12	15	38	17	33	26	20	29	26	9	6
17:00	66	53	42	56	66	53	43	21	12	24	11	6	12	26	23	32	19	20	25	31	13	17	41	20	29	31	22	31	29	14	7
18:00	58	53	51	66	79	45	46	25	18	27	13	7	15	30	26	11	18	19	28	25	14	19	40	22	31	40	25	41	32	20	7
19:00	61	52	67	86	69	43	54	36	32	36	18	10	17	30	36	14	19	17	33	29	20	25	51	31	27	44	27	40	40	23	9
20:00	75	49	108	90	73	45	56	44	54	52	26	13	27	33	41	15	17	19	35	42	25	50	63	42	28	52	28	53	49	32	11
21:00	75	57	126	106	77	61	61	68	68	65	29	15	33	34	39	15	15	17	34	53	39	53	87	58	38	66	26	43	70	45	18
22:00	75	56	158	118	95	81	66	87	79	70	36	13	48	39	35	16	15	16	34	60	54	85	91	59	39	79	26	42	75	42	27
23:00	73	58	174	121	103	102	66	82	86	77	38	14	54	49	39	15	18	17	31	62	66	106	96	65	47	83	22	54	75	36	34
24:00	71	57	185	138	105	108	66	91	101	90	33	15	57	51	37	13	18	17	31	63	69	104	97	75	52	87	20	56	68	38	28

Colors refer to the following air quality index (AQI) codes: dark green—very good; green—good; yellow—moderate; orange—average; red—bad; maroon—very bad.

**Table 2:** Daily and hourly PM10 content changes, with the hourly Polish AQI, average values for Kraków, March 2018 [39] (weekend marking in grey).

PM10																															
Day	1	2	3	4	5	6	7	8	9	10	11	12	13	14	15	16	17	18	19	20	21	22	23	24	25	26	27	28	29	30	31
Hour																															
01:00	79	94	74	213	188	133	125	83	118	125	147	48	18	70	57	60	17	31	24	41	81	85	148	130	109	73	110	32	64	71	46
02:00	88	100	73	215	182	127	120	82	116	126	137	58	16	72	58	52	15	28	24	41	83	75	145	124	109	73	109	29	66	65	38
03:00	110	103	76	213	181	135	121	81	112	120	125	42	8	68	59	48	15	27	23	38	89	66	147	125	108	74	107	30	58	62	39
04:00	119	108	76	216	169	136	110	77	100	112	115	32	12	70	52	52	16	25	24	36	99	61	141	124	114	76	98	33	58	53	38
05:00	121	109	89	213	164	128	112	75	94	112	110	28	9	67	42	52	15	23	22	41	104	58	139	129	119	87	95	36	52	51	36
06:00	130	110	93	198	157	122	113	81	100	113	110	24	13	66	38	48	15	23	21	46	91	60	129	126	119	92	90	45	48	56	35
07:00	146	115	103	195	172	118	100	84	95	115	104	28	19	75	34	46	19	28	22	55	46	68	129	139	115	100	99	53	52	62	35
08:00	170	116	106	184	178	116	112	84	88	118	101	38	31	70	30	46	20	30	23	49	17	69	123	127	111	116	107	59	55	51	41
09:00	143	130	102	161	177	123	130	70	43	121	86	68	30	71	27	45	21	31	27	40	19	67	130	121	96	109	104	60	65	46	43
10:00	86	118	90	128	207	126	140	54	32	117	74	71	30	39	25	49	20	36	29	42	29	52	82	99	73	98	79	67	47	34	31
11:00	56	120	83	98	214	106	129	48	26	72	50	71	33	35	27	51	21	35	26	42	37	35	58	78	59	60	45	59	34	26	41
12:00	64	112	79	102	171	80	105	35	38	61	36	51	25	35	34	51	22	33	31	56	35	33	58	69	56	52	43	52	19	25	36
13:00	84	112	74	85	143	81	57	31	35	44	30	40	27	34	40	50	23	33	31	63	28	33	59	48	52	45	41	47	24	20	42
14:00	94	119	77	83	121	82	49	27	32	34	29	42	27	26	37	43	24	39	30	49	25	30	58	40	45	39	42	43	26	20	40
15:00	101	106	69	81	110	72	49	26	32	36	23	29	27	20	38	44	27	36	31	55	29	32	59	33	43	36	33	48	38	21	33
16:00	101	93	68	84	98	70	53	30	28	42	25	34	34	29	37	40	26	30	39	36	25	29	62	29	46	44	26	48	48	25	24
17:00	95	84	59	88	108	71	59	34	29	48	24	27	32	33	38	36	25	33	37	39	33	31	61	31	49	54	29	50	55	29	25
18:00	101	82	84	109	123	54	65	43	38	55	36	20	39	36	47	17	24	29	37	35	30	48	64	36	61	64	42	60	64	32	19
19:00	107	81	108	135	108	55	73	57	62	69	44	27	39	37	60	16	23	28	43	35	41	93	81	49	51	72	52	62	78	35	18
20:00	112	70	143	152	113	63	77	66	98	92	73	27	51	41	57	18	22	29	48	47	56	148	101	59	43	83	46	62	94	39	18
21:00	104	77	173	168	118	77	78	101	116	137	91	32	60	41	58	19	28	31	48	60	78	151	116	71	55	90	38	56	101	49	32
22:00	93	71	199	165	136	95	74	115	117	142	83	32	77	43	46	21	23	26	51	68	87	163	123	85	66	105	33	55	105	51	30
23:00	97	75	199	175	133	109	75	118	135	136	65	37	81	50	46	21	26	28	47	74	93	156	132	89	62	100	28	65	91	50	36
24:00	98	77	204	186	131	116	76	118	139	145	54	30	79	53	51	16	33	30	41	76	90	149	138	103	68	122	28	64	76	48	35

Colors refer to the following air quality index (AQI) codes: dark green—very good; green—good; yellow—moderate; orange—average; red—bad; maroon—very bad.

**Table 3:** Daily and hourly C_6_H_6_ content changes, with the hourly Polish AQI, average values for Kraków, March 2018 [39] (weekend marking in grey).

C_6_H_6_																															
Day	1	2	3	4	5	6	7	8	9	10	11	12	13	14	15	16	17	18	19	20	21	22	23	24	25	26	27	28	29	30	31
Hour																															
01:00	5.6	5.5	3.2	11.4	11.4	9.1	10.3	5.1	8.8	10.2	9.6	5.7	0.7	4.4	3.4	4.6	1.0	0.8	0.8	1.6	4.5	4.8	11.2	8.2	5.5	3.9	4.7	1.1	5.0	4.8	4.7
02:00	6.1	5.8	3.2	14.9	11.7	10.0	10.3	5.1	9.6	9.9	9.3	4.1	0.5	4.2	3.5	3.7	0.9	0.7	0.8	1.8	4.7	4.2	11.8	8.0	5.7	3.9	4.7	1.1	3.5	5.5	5.5
03:00	7.4	6.0	3.3	12.3	10.9	11.0	9.4	4.8	8.9	9.1	9.3	2.9	0.4	4.3	3.9	3.8	0.8	0.6	0.8	1.7	4.8	3.7	12.3	8.3	5.8	3.9	4.7	1.1	3.8	4.3	5.8
04:00	8.2	6.7	3.4	12.4	12.2	10.9	11.0	4.9	9.1	9.0	8.9	1.7	0.4	3.9	3.0	3.7	0.7	0.6	0.8	1.6	5.0	3.2	11.8	8.4	6.4	3.9	4.1	1.3	2.5	2.7	5.3
05:00	8.1	6.7	3.6	11.3	11.5	10.1	11.6	5.2	8.0	9.0	8.9	1.2	0.6	3.8	2.3	3.3	0.6	0.6	0.8	1.6	5.4	2.9	10.6	9.1	7.4	4.2	4.2	1.7	2.7	2.9	5.0
06:00	8.0	6.9	3.9	10.4	9.6	9.2	12.4	5.3	8.3	9.0	8.7	1.1	1.1	3.6	1.9	2.6	0.6	0.6	1.0	1.9	4.8	2.9	10.2	7.6	7.0	4.7	4.5	2.4	2.1	4.1	3.5
07:00	9.5	6.5	4.6	9.6	10.2	8.6	11.7	6.6	8.4	9.3	8.4	1.6	1.8	3.9	2.0	2.5	0.7	0.6	1.1	2.5	3.5	3.1	9.3	7.6	7.2	5.6	5.1	3.3	1.7	3.9	4.4
08:00	11.6	6.5	5.1	9.2	12.1	7.6	12.7	6.2	7.3	9.5	7.9	1.8	2.2	3.9	2.1	3.5	0.8	0.6	1.1	2.4	2.0	2.6	8.2	6.8	6.1	5.2	5.2	2.5	3.2	2.6	6.2
09:00	8.6	7.3	5.3	6.8	18.9	8.0	12.9	4.7	3.6	8.6	5.2	2.6	1.3	3.0	2.1	3.9	0.9	0.7	1.2	2.2	0.9	2.1	5.7	7.7	4.2	4.4	4.9	2.1	4.2	1.4	4.0
10:00	4.5	5.8	3.7	5.4	12.4	7.5	11.3	2.6	2.5	5.6	3.7	1.6	0.9	2.1	1.8	3.7	0.9	0.7	1.4	2.1	0.7	1.3	3.2	3.5	3.1	3.2	3.7	2.4	3.1	0.8	3.2
11:00	3.2	4.9	3.1	4.0	9.9	5.3	8.0	1.9	1.6	3.1	2.4	1.5	0.7	1.5	2.0	3.7	1.0	0.7	1.3	2.0	0.7	0.9	2.1	3.4	2.6	2.9	2.7	1.8	1.5	0.7	3.0
12:00	2.8	4.5	2.9	2.9	7.9	4.3	3.2	1.3	1.1	2.5	1.5	0.8	0.6	1.9	1.9	3.8	1.0	0.8	1.3	2.1	0.7	0.8	1.7	3.2	2.5	2.2	2.6	1.3	1.2	0.7	4.0
13:00	2.9	4.5	2.8	2.7	8.0	4.2	1.7	1.1	0.8	1.9	1.2	0.5	0.7	1.9	1.7	3.8	0.8	0.8	1.4	2.3	0.7	0.8	1.9	2.2	2.1	1.6	2.1	1.1	1.1	0.7	2.1
14:00	2.8	4.3	3.0	2.6	7.8	4.5	1.9	1.1	0.9	1.2	1.2	0.5	0.6	1.5	1.4	3.6	1.0	0.9	1.4	2.4	0.7	0.9	1.8	1.7	1.9	1.5	1.5	1.2	1.2	0.8	1.9
15:00	3.1	4.3	2.5	2.4	5.0	4.9	2.1	1.2	0.9	1.4	1.2	0.5	0.8	1.6	1.6	4.3	1.0	1.3	1.6	2.0	0.9	0.9	1.9	1.3	2.0	1.6	1.4	1.3	1.3	0.8	0.8
16:00	3.1	3.4	2.5	9.2	4.4	8.2	2.3	1.7	0.8	1.7	1.0	0.6	1.0	2.4	1.5	3.0	1.0	1.0	1.9	2.2	0.9	1.0	1.9	1.3	1.9	1.9	1.5	1.4	1.3	0.8	0.7
17:00	3.1	3.2	2.6	3.3	9.1	5.5	2.7	1.8	1.1	5.1	1.0	0.9	1.2	2.6	1.5	2.7	1.0	0.9	2.0	2.4	1.0	1.2	2.1	1.4	1.9	2.3	1.5	3.7	2.2	1.1	1.0
18:00	3.1	3.4	2.9	3.3	7.8	5.7	3.1	2.1	1.5	3.9	1.7	1.0	1.2	2.6	2.2	2.5	1.1	1.0	2.1	2.3	1.1	1.5	2.3	1.9	2.2	2.7	1.6	3.5	2.0	1.3	1.2
19:00	3.6	3.3	4.2	3.9	6.9	5.2	4.2	2.5	2.6	3.4	2.4	1.1	1.8	2.6	4.5	2.1	1.2	1.1	2.0	2.5	1.8	3.1	3.2	3.4	2.3	3.4	2.0	3.2	2.9	3.1	1.4
20:00	4.7	3.4	7.4	5.9	7.4	5.5	4.9	3.5	4.7	5.1	3.3	1.4	2.3	2.5	4.7	1.9	1.0	1.1	1.7	3.1	2.1	5.2	4.2	4.6	2.3	4.1	1.9	3.1	4.6	2.9	2.5
21:00	5.7	3.5	10.3	10.6	7.2	6.6	5.5	4.9	5.7	6.5	3.8	1.3	2.8	2.6	3.7	1.7	1.0	1.0	1.9	4.1	3.1	6.9	4.4	4.6	2.9	4.7	1.6	3.3	6.5	3.3	2.5
22:00	5.6	3.5	12.9	14.3	7.5	8.4	5.5	6.7	7.4	7.1	4.3	1.3	4.0	2.9	3.0	1.7	1.0	1.1	1.7	4.4	4.2	9.4	6.3	6.0	3.2	5.2	1.3	4.4	6.9	3.4	3.1
23:00	5.0	3.6	13.2	8.3	8.4	9.2	5.1	7.2	7.9	9.1	3.7	1.3	4.6	3.6	3.5	1.4	0.9	0.9	1.7	4.3	4.8	11.3	6.9	4.4	3.4	5.0	1.0	3.7	6.1	4.7	3.7
24:00	4.7	3.5	14.1	9.8	8.8	9.9	5.1	8.8	8.3	9.7	2.8	1.2	4.4	3.3	3.8	1.3	0.9	0.9	1.7	4.4	5.3	11.5	7.1	5.1	3.6	5.0	1.1	3.5	4.9	4.9	3.0

Colors refer to the following air quality index (AQI) codes: dark green—very good; green—good; yellow—moderate; orange—average; red—bad; maroon—very bad.

**Table 4:** Daily and hourly NO_2_ content changes, with the hourly Polish AQI, average values for Kraków, March 2018 [39] (weekend marking in grey).

NO_2_																															
Day	1	2	3	4	5	6	7	8	9	10	11	12	13	14	15	16	17	18	19	20	21	22	23	24	25	26	27	28	29	30	31
Hour																															
01:00	44	30	27	80	80	82	59	57	61	67	59	26	22	55	36	29	14	11	10	16	41	44	70	63	71	51	49	23	32	46	31
02:00	54	32	29	75	72	75	62	53	53	64	55	26	24	49	36	26	12	10	10	16	42	36	60	55	63	53	43	22	34	45	23
03:00	58	30	30	70	62	74	59	47	44	55	52	21	18	44	35	24	11	9	9	15	41	36	53	53	56	49	41	27	28	47	29
04:00	56	32	27	64	56	72	56	43	40	49	45	18	25	42	33	25	9	8	12	19	46	38	51	46	54	51	41	34	33	41	29
05:00	55	34	46	62	61	72	61	40	42	46	37	18	32	45	27	25	9	9	12	20	49	44	47	45	50	57	51	51	35	47	33
06:00	60	40	57	60	66	74	63	47	47	46	36	36	49	48	26	28	10	7	18	38	56	58	53	58	49	64	65	61	55	58	35
07:00	71	50	67	58	83	81	67	63	56	47	36	52	72	58	36	30	13	9	28	47	52	70	63	63	46	64	73	65	67	61	37
08:00	79	53	64	56	93	76	71	76	64	56	40	66	73	63	31	31	16	10	30	44	42	68	68	55	51	74	65	58	72	56	38
09:00	69	52	58	55	104	62	79	73	71	64	45	70	51	64	26	31	20	11	31	49	33	58	74	51	46	78	59	53	80	45	41
10:00	46	43	42	51	104	55	86	57	58	55	36	53	37	49	34	32	22	11	35	43	27	43	63	51	34	70	50	45	67	34	30
11:00	31	38	35	38	85	52	83	37	43	43	37	50	31	41	31	34	21	13	32	38	26	28	41	42	29	49	47	36	56	27	32
12:00	32	37	35	38	75	52	64	32	28	42	28	42	26	41	32	35	23	12	32	47	26	28	41	42	31	45	48	31	36	27	32
13:00	33	39	34	35	71	53	38	30	27	38	27	22	26	43	38	41	21	14	33	46	27	31	39	36	30	48	49	31	35	26	32
14:00	37	46	36	31	66	60	36	32	27	33	29	25	32	36	40	43	22	14	32	46	26	33	43	34	28	48	42	34	37	27	29
15:00	46	48	33	36	67	74	40	35	28	36	27	31	34	34	31	48	19	18	36	42	32	36	46	30	32	43	38	39	38	31	29
16:00	45	45	37	43	68	80	49	39	35	44	32	41	42	46	32	53	20	17	39	43	30	40	50	29	33	48	38	36	45	34	26
17:00	45	43	34	47	79	74	50	48	36	50	29	40	47	47	36	54	19	17	37	53	38	44	45	32	42	62	40	38	52	34	31
18:00	49	47	54	57	104	75	61	56	57	49	43	42	52	46	40	33	21	18	42	58	40	74	51	38	57	67	47	48	66	39	47
19:00	52	45	88	75	83	77	69	69	79	66	57	40	55	49	45	27	19	18	36	70	53	96	55	44	68	70	51	45	74	40	41
20:00	53	35	110	77	83	79	69	73	100	81	85	44	62	50	47	24	20	20	35	81	65	113	60	48	66	71	39	42	78	62	36
21:00	48	33	111	77	95	86	66	87	94	90	81	48	65	48	41	21	17	21	31	68	85	116	72	52	75	66	32	34	73	70	31
22:00	47	33	98	84	105	77	64	88	93	84	75	39	71	47	30	21	16	16	30	57	84	99	77	60	62	61	26	36	71	67	40
23:00	44	33	87	81	98	62	61	82	83	76	56	46	70	45	28	22	13	15	25	61	72	86	74	59	59	53	22	42	61	49	45
24:00	36	31	82	84	91	58	58	69	81	70	35	45	61	41	28	17	12	13	20	49	60	75	68	70	57	49	22	35	46	36	40

Colors refer to the following air quality index (AQI) codes: dark green—very good; green—good; yellow—moderate; orange—average; red—bad; maroon—very bad.

**Table 5:** Daily and hourly O_3_ content changes, with the hourly Polish AQI, average values for Kraków, March 2018 [39] (weekend marking in grey).

O_3_																															
Day	1	2	3	4	5	6	7	8	9	10	11	12	13	14	15	16	17	18	19	20	21	22	23	24	25	26	27	28	29	30	31
Hour																															
01:00	14	44	75	5	6	3	3	4	2	4	4	56	66	4	6	20	67	80	86	70	42	21	5	5	3	3	8	35	35	3	38
02:00	4	43	75	4	19	3	2	12	3	4	2	49	68	5	5	18	71	83	85	71	32	12	5	5	4	3	12	31	36	3	39
03:00	3	41	69	5	40	2	3	5	3	3	3	48	69	3	6	16	74	84	86	71	30	23	4	4	3	2	8	21	36	14	37
04:00	3	39	67	6	42	3	2	9	4	4	3	51	62	3	5	13	73	82	84	59	18	21	5	4	2	2	15	21	30	23	37
05:00	4	37	8	3	37	3	3	6	6	4	3	65	43	3	21	15	66	80	85	57	12	10	5	3	5	3	24	11	25	6	37
06:00	3	34	3	4	33	3	2	1	3	3	4	63	44	3	27	9	72	79	82	44	8	5	5	2	7	3	8	3	8	2	34
07:00	4	28	4	4	12	2	3	3	3	4	3	51	6	3	20	8	72	79	78	37	11	8	4	2	4	4	4	7	5	3	32
08:00	5	27	6	4	5	4	3	3	4	3	8	50	15	3	16	9	70	79	72	31	43	19	11	27	11	5	2	23	6	15	34
09:00	9	29	26	32	8	11	3	10	9	5	23	40	33	8	18	10	69	77	75	38	66	53	17	61	38	16	6	25	3	46	27
10:00	34	43	62	52	16	30	5	30	28	15	35	52	50	27	21	14	70	78	78	59	77	75	37	77	62	27	7	29	32	70	28
11:00	57	59	73	63	37	46	27	53	60	32	54	61	61	30	38	16	69	79	80	64	82	78	63	79	74	61	10	61	47	78	27
12:00	64	72	77	71	54	59	60	66	71	49	76	63	69	31	53	17	71	82	81	65	83	78	64	83	83	80	13	67	61	88	32
13:00	73	81	86	86	74	61	68	69	77	64	84	77	71	21	50	18	72	82	84	62	84	80	65	95	99	75	20	70	58	91	41
14:00	77	87	88	90	92	58	71	67	81	78	89	76	73	14	43	18	77	85	84	56	82	86	66	93	104	75	32	74	31	91	56
15:00	65	91	96	96	93	54	65	65	81	79	91	77	67	13	41	20	78	83	84	67	78	83	59	95	107	77	33	77	37	93	75
16:00	66	90	96	95	91	45	50	59	76	80	91	71	59	13	39	21	77	86	84	66	78	78	56	98	107	69	38	76	40	94	78
17:00	64	89	96	98	64	30	47	52	68	71	86	64	55	7	32	10	80	85	83	55	75	73	62	96	98	57	36	74	34	87	74
18:00	62	84	85	79	21	15	25	35	43	69	72	60	44	7	22	12	80	84	80	59	67	48	59	93	70	44	39	67	16	79	59
19:00	53	81	47	70	46	11	13	18	10	49	68	55	37	8	8	21	76	84	79	46	46	8	44	83	60	35	26	57	4	74	59
20:00	46	86	5	68	46	5	22	11	4	13	30	57	19	5	6	31	77	85	80	28	32	6	34	66	30	28	28	55	4	51	59
21:00	46	80	5	58	41	2	24	3	3	3	18	58	12	9	12	42	81	84	79	23	12	5	10	66	7	26	43	55	3	5	51
22:00	43	78	4	27	6	3	12	3	5	4	42	60	4	6	20	52	84	84	79	31	6	6	6	61	14	25	48	52	3	4	39
23:00	40	77	5	35	4	4	6	3	3	3	65	44	3	7	17	55	82	84	81	25	6	5	6	58	15	11	51	47	3	17	10
24:00	41	78	5	15	4	3	6	2	3	4	68	54	3	8	21	62	79	84	76	26	8	5	6	19	3	19	55	39	3	30	20

Colors refer to the following air quality index (AQI) codes: dark green—very good; green—good; yellow—moderate; orange—average; red—bad; maroon—very bad.

**Table 6:** Enrichment index classes for deposited PM in Kraków (modified after [49]). I_geo_: geoaccumulation index, CF: contamination factor, EF: enrichment factor, ERI: ecological risk index, mHQ: modified hazard quotient.

Element	I_geo_		CF		EF (mean)		ERI		mHQ	
	Value	Class	Value	Class	Value	Class	Value	Class	Value	Class
As	5.6	6	3.06	considerable	2.63	minor	30.6	low	1.90	moderate severity
As (PM10)	2.1	3	0.27	low	0.23	no	2.7	low	0.17	nil to very low
Ba	15.5	6	0.18	low	0.15	no	-	-	-	-
Be	−0.4	0	0.26	low	0.22	no	-	-	-	-
Cd	−3.8	0	13.3	very high	11.44	severe	400	very high	1.24	low severity
Cd (PM10)	−4.6	0	7.56	very high	6.49	moderately severe	227	high	0.70	very low
Co	4.8	5	0.14	low	0.12	no	0.7	low	-	-
Cr	12.4	6	0.98	low	0.84	no	2.0	low	2.12	considerable severity
Cu	10.4	6	2.66	moderate	2.28	minor	13.3	low	1.57	moderate severity
Li	8.2	6	1.00	moderate	0.86	no	-	-	-	-
Mn	18.3	6	0.81	low	0.69	no	-	-	-	-
Ni	9.6	6	0.52	low	0.45	no	2.6	low	1.18	low severity
Ni (PM10)	5.7	6	0.04	low	0.03	no	0.2	low	0.08	nil to very low
Pb	9.9	6	5.03	considerable	4.32	moderate	25.1	low	1.86	moderate severity
Pb (PM10)	−1.3	0	0.002	low	0.002	no	0.01	low	0.001	nil to very low
Sn	4.1	5	5.95	considerable	5.11	moderately severe	-	-	-	-
Sr	14.9	6	0.44	low	0.38	no	-	-	-	-
Ti	19.6	6	0.04	low	0.03	no	-	-	-	-
Tl	−2.7	0	0.29	low	0.25	no	2.9	low	-	-
V	10.9	6	0.31	low	0.27	no	-	-	-	-
Zn	18.0	6	86.87	very high	74.56	extremely severe	86.9	considerable	36.45	extreme severity

−: not applicable.

**Table 7:** Risk assessment values for the residents of Kraków.

Pollutant	Inhalation				Ingestion				Dermal contact			
	HQ		CR		HQ		CR		HQ		CR	
	Adult	Child	Adult	Child	Adult	Child	Adult	Child	Adult	Child	Adult	Child
**Ambient Air**												
NO_2_	1.13 × 10^0^	2.64 × 10^0^	na	na	–				–			
Benzen	1.02 × 10^−1^	1.02 × 10^−1^	8.21 × 10^−12^	2.05 × 10^−12^								
PM2.5	7.23 × 10^0^	7.23 × 10^0^	na	na								
PM10	1.42 × 10^0^	3.32 × 10^0^	na	na								
Pb (PM10)	2.90 × 10^−3^	6.76 × 10^−3^	2.67 × 10^−5^	8.52 × 10^−8^								
As (PM10)	8.31 × 10^−2^	8.31 × 10^−2^	1.84 × 10^−12^	4.59 × 10^−13^								
Cd (PM10)	6.52 × 10^−2^	6.52 × 10^−2^	4.02 × 10^−13^	1.01 × 10^−13^								
Ni (PM10)	1.77 × 10^−2^	1.77 × 10^−2^	1.42 × 10^−16^	3.55 × 10^−17^								
BaP (PM10)	2.83 × 10^0^	2.83 × 10^0^	1.16 × 10^−12^	2.91 × 10^−13^								
BaA (PM10)	na	na	1.15 × 10^−13^	2.88 × 10^−14^								
BbF (PM10)	na	na	6.65 × 10^−14^	1.66 × 10^−14^								
BjF (PM10)	na	na	1.06 × 10^−13^	2.66 × 10^−14^								
BkF (PM10)	na	na	5.31 × 10^−15^	1.33 × 10^−15^								
DBahA (PM10)	na	na	1.72 × 10^−13^	4.29 × 10^−14^								
**Deposited PM**												
Al	2.15 × 10^−2^	2.15 × 10^−2^	na	na	8.70 × 10^−3^	8.12 × 10^−2^	na	na	3.67 × 10^−2^	1.93 × 10^−1^	na	na
As	1.65 × 10^−2^	1.65 × 10^−2^	1.88 × 10^−18^	4.52 × 10^−19^	3.96 × 10^−2^	3.51 × 10^−1^	6.11 × 10^−6^	1.35 × 10^−5^	8.47 × 10^−3^	4.44 × 10^−2^	1.31 × 10^−6^	1.71 × 10^−6^
Ba	3.77 × 10^−3^	3.77 × 10^−3^	na	na	7.42 × 10^−4^	6.29 × 10^−3^	na	na	3.22 × 10^−3^	1.69 × 10^−2^	na	na
Be	4.57 × 10^−4^	4.57 × 10^−4^	2.56 × 10^−21^	6.13 × 10^−22^	3.55 × 10^−4^	2.88 × 10^−3^	na	na	1.56 × 10^−3^	8.19 × 10^−3^	na	na
Cd	1.95 × 10^−3^	1.95 × 10^−3^	1.16 × 10^−20^	2.78 × 10^−21^	1.49 × 10^−3^	1.16 × 10^−2^	na	na	6.65 × 10^−6^	3.49 × 10^−5^	na	na
Co	6.63 × 10^−3^	6.63 × 10^−3^	4.85 × 10^−20^	1.16 × 10^−20^	1.00 × 10^−2^	7.51 × 10^−2^	na	na	4.53 × 10^−2^	2.38 × 10^−1^	na	na
Cr(III)	1.21 × 10^−8^	2.82 × 10^−8^	na	na	7.57 × 10^−5^	5.48 × 10^−4^	na	na	3.47 × 10^−4^	1.82 × 10^−3^	na	na
Cr(VI)	1.52 × 10^−2^	1.52 × 10^−2^	7.11 × 10^−17^	1.70 × 10^−17^	3.74 × 10^−2^	2.62 × 10^−1^	1.92 × 10^−5^	3.36 × 10^−5^	1.74 × 10^−1^	9.10 × 10^−1^	8.92 × 10^−5^	1.17 × 10^−4^
Cu	6.27 × 10^−7^	1.46 × 10^−6^	na	na	3.83 × 10^−3^	2.60 × 10^−2^	na	na	1.80 × 10^−2^	9.44 × 10^−2^	na	na
Fe	1.16 × 10^−5^	2.72 × 10^−5^	na	na	7.01 × 10^−2^	4.62 × 10^−1^	na	na	3.34 × 10^−1^	1.75 × 10^0^	na	na
Li	2.13 × 10^−6^	4.96 × 10^−6^	na	na	1.26 × 10^−2^	8.09 × 10^−2^	na	na	6.10 × 10^−2^	3.20 × 10^−1^	na	na
Mn	2.11 × 10^−1^	2.11 × 10^−1^	na	na	3.08 × 10^−2^	1.92 × 10^−1^	na	na	1.50 × 10^−1^	7.88 × 10^−1^	na	na
Ni	4.60 × 10^−3^	4.60 × 10^−3^	5.25 × 10^−18^	1.26 × 10^−18^	1.43 × 10^−3^	8.68 × 10^−3^	na	na	7.07 × 10^−5^	3.71 × 10^−4^	na	na
Pb	4.92 × 10^−6^	1.15 × 10^−5^	2.48 × 10^−10^	1.45 × 10^−10^	2.82 × 10^−2^	1.67 × 10^−1^	1.42 × 10^−6^	2.11 × 10^−6^	1.41 × 10^−1^	7.41 × 10^−1^	7.12 × 10^−6^	9.34 × 10^−6^
Sn	4.20 × 10^−9^	9.79 × 10^−9^	na	na	2.38 × 10^−5^	1.38 × 10^−4^	na	na	1.21 × 10^−4^	6.32 × 10^−4^	na	na
Sr	4.72 × 10^−8^	1.10 × 10^−7^	na	na	2.64 × 10^−4^	1.50 × 10^−3^	na	na	1.35 × 10^−3^	7.10 × 10^−3^	na	na
V	5.08 × 10^−3^	5.08 × 10^−3^	na	na	6.69 × 10^−3^	3.71 × 10^−2^	na	na	3.47 × 10^−2^	1.82 × 10^−1^	na	na
Zn	3.91 × 10^−6^	9.12 × 10^−6^	na	na	2.14 × 10^−2^	1.16 × 10^−1^	na	na	1.12 × 10^−1^	5.89 × 10^−1^	na	na
Zr	1.15 × 10^−5^	2.67 × 10^−5^	na	na	6.20 × 10^−2^	3.31 × 10^−1^	na	na	3.29 × 10^−1^	1.73 × 10^0^	na	na
Total	1.32 × 10^+1^	1.66 × 10^+1^	2.67 × 10^−5^	8.53 × 10^−8^	3.36 × 10^−1^	2.21 × 10^0^	2.67 × 10^−5^	4.93 × 10^−5^	1.45 × 10^0^	7.61 × 10^0^	9.77 × 10^−5^	1.28 × 10^−4^
Adult total HQ		1.50 × 10^+1^										
Child total HQ		2.64 × 10^+1^										
Adult total CR		1.51 × 10^−4^										
Child total CR		1.77 × 10^−4^										

na: not available, due to missing toxicological data; –: not applicable to the available data.

## Supplementary Materials

The following are available online at https://www.mdpi.com/1660-4601/17/17/6063/s1, Figure S1. A demonstrative wind rose in Kraków Poland: share of wind directions in % (a), and wind speed distribution in m/s (b), divided into cool and warm half-years. Figure S2. Location of air-flow corridors and Regional Environmental Protection Inspectorate air monitoring stations in Kraków. Table S1. Description of the air monitoring stations operated by the Regional Environmental Protection Inspectorate (REPI) in Kraków. Table S2. Ranges of the 1 h concentrations in the Polish air quality index (AQI) for selected pollutants. Table S3. The Polish AQI index and the corresponding health recommendations for residents. Table S4. Description of the enrichment indices used in the study. Table S5. Exposure parameters used for the risk assessment calculations under resident scenario in the study. Table S6. Toxicological parameters used for the risk assessment calculations under resident scenario in the study. Table S7. Changes in annual air pollutant contents in Kraków in 2005–2020, with permissible levels. Table S8. Changes in monthly air pollutant contents in 2018 in Kraków; average values of all the monitoring stations. Table S9. Changes in daily air pollutant contents in a selected winter month, average values for Kraków, March 2018, with recommended concentrations. Table S10. Changes in daily air pollutant contents in a selected summer month, average values for Kraków, July 2018, with recommended concentrations. Table S11. Daily and hourly SO_2_ content changes, with the hourly Polish AQI index, average values for Kraków, March 2018. Table S12. Daily and hourly CO content changes, with the hourly Polish AQI index, average values for Kraków, March 2018. Table S13. Changes in monthly air-pollutant contents in the first half of 2020 in Kraków, average values from all the monitoring stations. Table S14. Estimated daily intake values for the resident of Kraków, in reference to exposure pathways.

## References

[1] Nowicki M., Ribbe L. (2001). Problems of Eco-Development in Poland.

[2] Manecki A. (2015). Alphabet of Memories: About People of Science and Events of the Past.

[3] Kleczkowski P. (2019). Smog in Poland. Causes, Effects, Prevention.

[4] World Health Organization (2016). WHO Ambient Air Pollution Database May 2016.

[5] Polish City Registers Second Worst Air Pollution in the World as Smog Descends on Poland, Notes from Poland. https://notesfrompoland.com/2020/01/17/polish-city-registers-second-worst-air-pollution-in-the-world-as-smog-descends-on-poland/.

[6] Jedrychowski W., Maugeri U., Jedrychowska-Bianchi I., Flak E. (2005). Effect of indoor air quality in the postnatal period on lung function in pre-adolescent children: A retrospective cohort study in Poland. Public Health.

[7] Jedrychowski W.A., Perera F.P., Spengler J.D., Mroz E., Stigter L., Flak E., Majewska R., Klimaszewska-Rembiasz M., Jacek R. (2013). Intrauterine exposure to fine particulate matter as a risk factor for increased susceptibility to acute broncho-pulmonary infections in early childhood. Int. J. Hyg. Environ. Health.

[8] Adamiec E., Jarosz-Krzemińska E., Wieszała R. (2016). Heavy metals from non-exhaust vehicle emissions in ur-ban and motorway road dusts. Environ. Monit. Assess..

[9] Biuletyn Informacji Publicznej Urząd Marszałkowski Województwa Małopolskiego. https://bip.malopolska.pl/umwm,a,1283900,uchwala-nr-xxxii45217-sejmiku-wojewodztwa-malopolskiego-z-dnia-23-styczna-2017-r-w-sprawie-wprowadze.html..

[10] Bokwa A. (2008). Environmental impact of long-term air pollution changes in Krakow, Poland. Polish J. of Environ. Stud..

[11] Samek L. (2009). Chemical characterization of selected metals by X-ray fluorescence method in particulate matter collected in the area of Krakow, Poland. Microchem. J..

[12] Wilczyńska-Michalik W., Michalik M. (2015). Composition and origin of dust particles in atmosphere in Kraków. Aura.

[13] Wilczyńska-Michalik W., Pietras B., Samek L., Furman L., Łatkiewicz A., Rzeźnikiewicz K., Michalik M. (2015). Submicrometer particles in air pollution in Kraków. Aura.

[14] Choi H., Melly S.J., Spengler J. (2015). Intraurban and Longitudinal Variability of Classical Pollutants in Kraków, Poland, 2000–2010. Int. J. Environ. Res. Public Health.

[15] Samek L., Stegowski Z., Furman L., Fiedor J. (2016). Chemical content and estimated sources of fine fraction of particulate matter collected in Krakow. Air. Qual. Atmos. Health.

[16] Samek L. (2016). Overall human mortality and morbidity due to exposure to air pollution. Int. J. Occup. Med. Environ. Health.

[17] Bogacki M., Bździuch P. (2019). Predicting the Spatial Distribution of Emissions from Urban Buses Based on Previ-ously Measured Data and Scenarios for Their Modernization in the Future. Case Study: Kraków, Poland. Atmos. Environ..

[18] Rzeszutek M., Bogacki M., Bździuch P., Szulecka A. (2019). Improvement assessment of the OSPM model per-formance by considering the secondary road dust emissions. Transport. Res. D Tr. E..

[19] Biuletyn Informacji Publicznej (2013). Change in the Study of Conditions and Directions for Spatial Development of the City of Krakow.

[20] Ośródka L., Godłowska J., Hajto M., Rozwoda W., Wojtylak M. (2010). Determining the Anemological Conditions for the Area of Kraków on the Basis of Data from the Institute of Meteorology and Water Management Observation and Measurement Network.

[21] Bini C., Bech J. (2014). PHEs, Environment and Human Health-Potentially Harmful Elements in the Environment and the Impact on Human Health.

[22] Peters A., Dockery D.W., Muller J.E., Mittleman M.A. (2001). Increased particulate air pollution and the triggering of myocardial infarction. Circulation.

[23] Brunekreef B., Holgate S.T. (2002). Air pollution and health. Lancet.

[24] Pope C.A., Burnett R.T., Thun M.J., Calle E.E., Krewski D., Ito K., Thurston G.D. (2002). Lung cancer, car-diopulmonary mortality and long-term exposure to fine particulate air pollution. JAMA.

[25] Rajagopalan S., Al-Kindi S.G., Brook R.D. (2018). Air pollution and cardiovascular disease. J. Am. Coll. Cardiol..

[26] Samet J.M., Dominici F., Curriero F.C., Coursac I., Zeger S.L. (2000). Fine particulate air pollution and mortality in 20 U.S. Cities, 1987–1994. New Engl. J. Med..

[27] Polichetti G., Cocco S., Spinali A., Trimarco V., Nunziata A. (2009). Effects of particulate matter (PM10, PM2.5 and PM1) on the cardiovascular system. Toxicology.

[28] Lippmann M., Chen L.C., Gordon T., Ito K., Thurston G.D. (2013). National particle component toxicity (NPACT) Initiative: Integrated epidemiologic and toxicologic studies of the health effects of particulate matter components. Res Rep. Health Eff. Inst..

[29] Wang M., Beelen R., Stafoggia M., Raaschou-Nielsen O., Andersen Z.J., Hoffmann B., Fischer P., Houthuijs D., Nieuwenhuijsen M., Weinmayr G. (2014). Long-term exposure to elemental constituents of particulate matter and cardiovascular mortality in 19 European cohorts: Results from the ESCAPE and TRANSPHORM projects. Environ. Int..

[30] Chau T.-T., Wang K.-Y. (2020). An association between air pollution and daily most frequently visits of eighteen outpatient diseases in an industrial city. Sci. Rep..

[31] Zuśka Z., Kopcińska J., Dacewicz E., Skowera B., Wojkowski J., Ziernicka-Wojtaszek A. (2019). Application of the Principal Component Analysis (PCA) method to assess the impact of meteorological elements on con-centrations of particulate matter (PM10): A case study of the Mountain Valley (the Sącz Basin, Poland). Sustainability.

[32] Kastury F., Smith E., Juhasz A.L. (2017). A critical review of approaches and limitations of inhalation bioavaila-bility and bioaccessibility of metal (loid)s from ambient particulate matter or dust. Sci. Total. Environ..

[33] Ahmad H.R., Mehmood K., Sardar M.F., Maqsood M.A., Rehman M.Z.U., Zhu C., Li H. (2019). Integrated risk assessment of potentially toxic elements and particle pollution in urban road dust of megacity of Pakistan. Hum. Ecol. Risk Assess. Int. J..

[34] Musa A., Hamza S.M., Kidak R. (2019). Street dust heavy metal pollution implication on human health in Nicosia, North Cyprus. Environ. Sci. Pollut. Res..

[35] Kadhum S.A. (2020). A preliminary study of heavy metals pollution in the sandy dust storms and its human risk assessment from middle and south of Iraq. Environ. Sci. Pollut. Res..

[36] Gruszecka-Kosowska A., Wdowin M. (2016). The mineralogy, geochemistry and health risk assessment of depos-ited particulate matter (PM) in Kraków, Poland. Geol. Geophys. Environ..

[37] Pachurka Ł., Gruszecka-Kosowska A., Kobus D., Sówka I. (2018). Assessment of inhalational exposure of resi-dents of Wroclaw, Kraków and Warszawa to benzo[a]pyrene. Ecological Chemistry and Engineering A—Chemia i Inżynieria Ekologiczna A.

[38] Gruszecka-Kosowska A. (2018). Assessment of the Kraków inhabitants’ health risk caused by the exposure to inhalation of outdoor air contaminants. Stoch. Env Res. Risk A.

[39] Regional Environmental Protection Inspectorate in Kraków, Poland Wojewódzki Inspektorat Ochrony Środowiska w Krakowie. http://Kraków.pios.gov.pl/.

[40] Regional Environmental Protection Inspectorate in Kraków (2006). Assessment of Air Quality in the Małopolska Voivodeship in 2005.

[41] Tarnów Branch (2006). Examination of Air Pollution with Benzene, by the Indicator Method, in Accordance with the Environmental Monitoring Program in the Małopolska Voivodeship in 2005. Report on Air Pollution Analysis in the Area of Małopolska Voivodeship in 2005.

[42] Regional Environmental Protection Inspectorate in Kraków (2006). Initial Assessment of Air Quality in the Małopolska Voivodeship in Terms of the Content of Arsenic, Cadmium, Mercury, Nickel and Benzo (a) Pyrene in PM10 Dust and Adjustment of the Assessment System to the Requirements of Directive 2004/107/EC.

[43] Regional Environmental Protection Inspectorate in Kraków (2007). Assessment of Air Quality in the Małopolska Voivodeship in 2006 (Verified).

[44] Tarnów Branch (2007). Examination of Air Pollution with Benzene, by the Indicator Method, in Accordance with the Environmental Monitoring Program in the Małopolska Voivodeship in 2006. Report on Air Pollution Analysis in the Area of Małopolska Voivodeship in 2006.

[45] Tarnów Branch (2008). Examination of Air Pollution with Benzene, by the Indicator Method, in Accordance with the Environmental Monitoring Program in the Małopolska Voivodeship for 2007–2009. Report on Air Pollution Analysis with Benzene in the Area of Małopolska Voivodeship in 2007.

[46] Biuletyn Informacji Publicznej (2012). Regulation of the Minister of the Environment Concerning the Levels of Certain SubStances in the Air of 24 August 2012.

[47] European Environment Agency (2019). Air quality in Europe—2019 Report. EEA Report No 10/2019.

[48] Goddard S.L., Williams K.R., Robins C., Butterfield D.M., Brown R.J.C. (2019). Concentration trends of metals in ambient air in the UK: A review. Environ. Monit. Assess..

[49] Gruszecka-Kosowska A. (2019). Deposited particulate matter enrichment in heavy metals and related health risk: A case study of Krakow, Poland. Proceedings.

[50] Kabata-Pendias A. (2011). Trace Elements in Soils and Plants.

[51] Rudnick R., Gao S. (2014). Composition of the Continental Crust. Treatise on Geochemistry.

[52] Benson N.U., Adedapo A.E., Fred-Ahmadu O.H., Williams A., Udosen E.D., Ayejuyo O.O., Olajire A.A. (2018). New ecological risk indices for evaluating heavy metals contamination in aquatic sediment: A case study of the Gulf of Guinea. Reg. Stud. Mar. Sci..

[53] Office of Emergency and Remedial Response (1989). Risk Assessment Guidance for Superfund: Human Health Evaluation Manual, Part. A.

[54] Office of Emergency and Remedial Response (2009). Risk Assessment Guidance for Superfund Volume I: Human Health Evaluation Manual Supplemental Guidance.

[55] Wcisło E., Bronder J., Bubak A., Rodríguez-Valdés E., Gallego J.L.R. (2016). Human health risk assessment in restoring safe and productive use of abandoned contaminated sites. Environ. Int..

[56] Ferreira-Baptista L., De De Miguel E. (2005). Geochemistry and risk assessment of street dust in Luanda, Angola: A tropical urban environment. Atm. Environ..

[57] Office of Solid Waste and Emergency Response (1991). Role of the Baseline Risk Assessment in Superfund Remedy Selection Decisions.

[58] Kanchan K., Gorai A.K., Goyal P. (2015). A Review on Air Quality Indexing System. Asian J. Atm. Environ..

[59] Environmental Monitoring Library (2007). Voivodeship Inspectorate for Environmental Protection in Kraków. Report on the State of the Environment in Małopolska in 2006.

[60] Yadav S., Kumbhar N., Jan R., Roy R., Satsangi P.G. (2018). Genotoxic effects of PM10 and PM2.5 bound metals: Metal bioaccessibility, free radical generation, and role of iron. Environ. Geochem. Health.

[61] Ministry of the Environment Republic of Poland (2013). Polish National Strategy for Adaptation to Climate Change (NAS 2020) with the Perspective by 2030.

[62] Wójcik R., Miętus M., Miȩtus M. (2014). Some features of long-term variability in air temperature in Poland (1951–2010). Przegląd Geograficzny.

[63] Stathopoulou E., Mihalakakou G., Santamouris M., Bagiorgas H.S. (2008). On the impact of temperature on tropospheric ozone concentration levels in urban environments. J. Earth Syst. Sci..

[64] Kumar A., Mishra R.K. (2018). Human health risk assessment of major air pollutants at transport corridors of Delhi, India. J. Transp. Health.

[65] (2020). Airly, Airly Maps. https://airly.eu/map/pl/.

[66] Chalvatzaki E., Chatoutsidou S.E., Lehtomäki H., Almeida S.M., Eleftheriadis K., Hänninen O., Lazaridis M. (2019). Characterization of human health risks from particulate air pollution in selected European cities. Atmophere.

[67] Zgłobicki W., Telecka M., Skupiński S. (2019). Assessment of short-term changes in street dust pollution with heavy metals in Lublin (E Poland)—Levels, sources and risks. Environ. Sci. Pollut. Res. Int..

[68] Tainio M., Juda-Rezler K., Reizer M., Warchałowski A., Trapp W., Skotak K. (2012). Future climate and adverse health effects caused by fine particulate matter air pollution: Case study for Poland. Reg. Environ. Chang..

[69] Li H.-H., Chen L.-J., Yu L., Guo Z.-B., Shan C.-Q., Lin J.-Q., Gu Y.-G., Yang Z.-B., Yang Y.-X., Shao J.-R. (2017). Pollution characteristics and risk assessment of human exposure to oral bio-accessibility of heavy metals via urban street dusts from different functional areas in Chengdu, China. Sci. Total. Environ..

[70] Men C., Liu R., Xu F., Wang Q., Guo L., Shen Z. (2018). Pollution characteristics, risk assessment, and source apportionment of heavy metals in road dust in Beijing, China. Sci. Total. Environ..

[71] Jahandari A. (2020). Pollution status and human health risk assessments of selected heavy metals in urban dust of 16 cities in Iran. Environ. Sci. Pollut. Res..

[72] Han X., Lu X., Zhang Q., Hai Q., Pan H., Wuyuntana (2016). Grain-size distribution and contamination characteristics of heavy metal in street dust of Baotou, China. Environ. Earth Sci..

