# Supplementary material for: The Condition of Air Pollution in Kraków, Poland, in 2005–2020, with Health Risk Assessment
Source: Int J Environ Res Public Health. 2020 Aug 20;17(17):6063. doi: 10.3390/ijerph17176063 (PMC7503758; doi:10.3390/ijerph17176063)
Supplement: Supplementary file 1 [file ijerph-17-06063-s001.pdf]

International Journal of
Environmental Research and Public Health

MDPI

Supplementary Materials

The condition of air pollution in Kraków, Poland, in 2005-2020, with health risk assessment

Paulina Traczyk and Agnieszka Gruszecka-Kosowska [1,*]

[1] AGH University of Science and Technology, Faculty of Geology, Geophysics and Environmental Protection, Department of Environmental Protection, Al. Mickiewicza 30, 30-059 Krakow, Poland
* Correspondence: agnieszka.gruszecka@agh.edu.pl (A.G.-K.), p.traczyk94@gmail.com (P.T.)

Figure S1. A demonstrative wind rose in Kraków Poland: share of wind directions in % (a), and wind speed distribution in m/s (b), divided into cool and warm half-years.

Figure S2. Location of air-flow corridors and Regional Environmental Protection Inspectorate air monitoring stations in Kraków.

Table S1. Description of the air monitoring stations operated by the Regional Environmental Protection Inspectorate (REPI) in Kraków.

Table S2. Ranges of the 1h concentrations in the Polish air quality index (AQI) for selected pollutants.

Table S3. The Polish AQI index and the corresponding health recommendations for residents.

Table S4. Description of the enrichment indices used in the study.

Table S5. Exposure parameters used for the risk assessment calculations under resident scenario in the study.

Table S6. Toxicological parameters used for the risk assessment calculations under resident scenario in the study.

Table S7. Changes in annual air pollutant contents in Kraków in 2005-2020, with permissible levels.

Table S8. Changes in monthly air pollutant contents in 2018 in Kraków; average values of all the monitoring stations.

Table S9. Changes in daily air pollutant contents in a selected winter month, average values for Kraków, March 2018, with recommended concentrations.

Table S10. Changes in daily air pollutant contents in a selected summer month, average values for Kraków, July 2018, with recommended concentrations.

Table S11. Daily and hourly SO_2 content changes, with the hourly Polish AQI index, average values for Kraków, March 2018.

Table S12. Daily and hourly CO content changes, with the hourly Polish AQI index, average values for Kraków, March 2018.

Table S13. Changes in monthly air-pollutant contents in the first half of 2020 in Kraków, average values from all the monitoring stations.

Table S14. Estimated daily intake values for the resident of Kraków, in reference to exposure pathways.

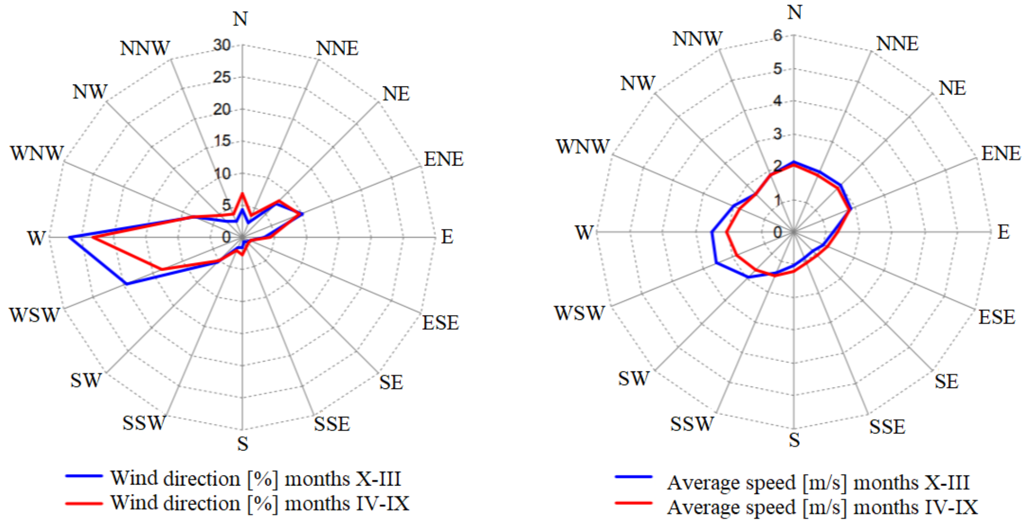

Figure S1. A demonstrative wind rose in Kraków Poland: share of wind directions in % (a), and wind speed distribution in m/s (b), divided into cool and warm half-years [1].

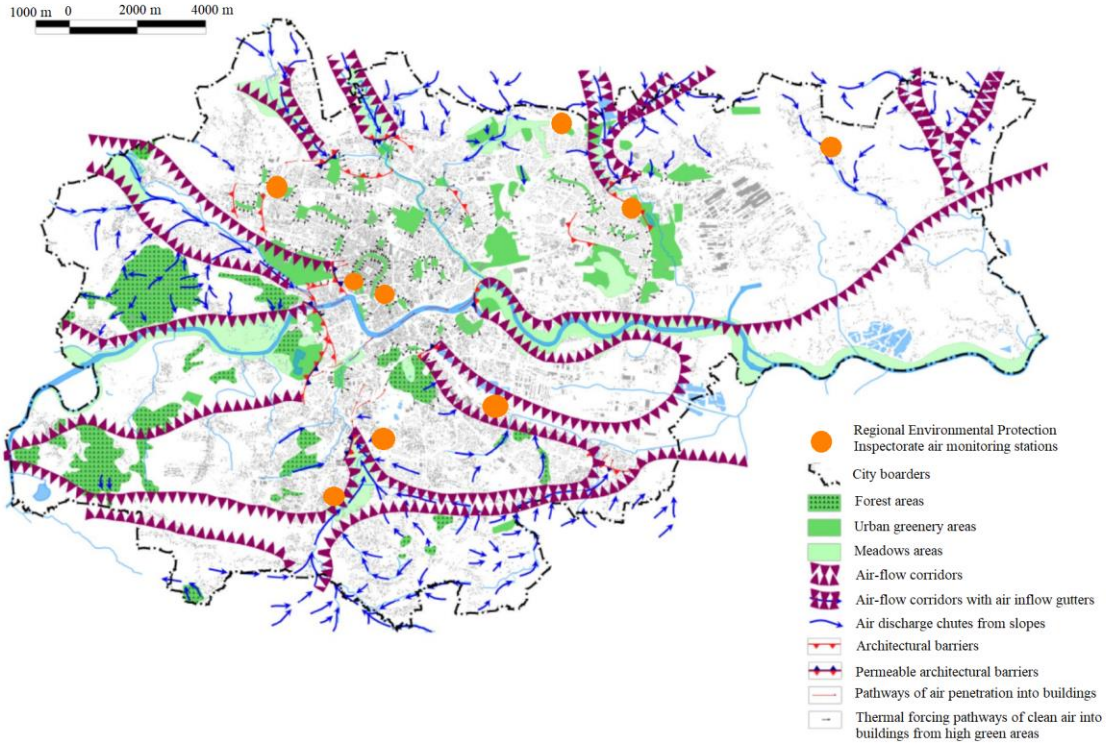

Figure S2. Location of air-flow corridors and Regional Environmental Protection Inspectorate air monitoring stations in Kraków (modified after [2]).

Table S1. Description of the air monitoring stations operated by the Regional Environmental Protection Inspectorate (REPI) in Kraków [3].

No.	Station address and Code	Station site*	Purpose	Measurement method and measured compounds
1	Aleja Krasińskiego (PL0012A)	Roadside	Public exposure assessment	Automatic: C_6H_6, CO, NO, NO_2, NO_x, PM10, PM2.5<br>Manual: C_6H_6, PM10, BaP(PM10)
2	Nowa Huta (PL0039A)	Industrial district	Public exposure assessment	Automatic: C_6H_6, CO, NO, NO_2, NO_x, SO_2, PM10, PM2.5<br>Manual: As(PM10), Cd(PM10), Ni(PM10), Pb(PM10), PM10, C_6H_6, BaP(PM10)
3	Kraków –Kurdwanów (PL0501A)	City	Public exposure assessment	Automatic: C_6H_6, NO, NO_2, NO_x, O_3, SO_2, PM10, PM2.5<br>Manual: As(PM10), Cd(PM10), Ni(PM10), Pb(PM10), PM10, PM2.5, C_6H_6, BaA(PM10), BaP(PM10), BbF(PM10), BjF(PM10), BkF(PM10), DBahA(PM10)
4	Kraków – ul.Dietla (MAL028)	Roadside	Population exposure assessment	Automatic: NO_2, NO_x, NO, PM10
5	Kraków – os.Piastów (MAL031)	City	Public exposure assessment	Automatic: PM10<br>Manual: PM10, BaP(PM10), PM2.5
6	Kraków – ul.Złoty Róg (MAL032)	City	Public exposure assessment	Automatic: PM10<br>Manual: PM10, BaP(PM10)
7	Kraków – os.Wadów (MAL040)	Industrial district	Public exposure assessment	Automatic: PM10<br>Manual: PM10, BaP(PM10), As(PM10), Cd(PM10), Pb(PM10),
8	Kraków – os.Swoszowice (PL0735A)	City	Protecting human health	Automatic: PM10<br>Manual: PM10, BaP(PM10)
9	Kraków – ul.Telimeny (MAL042) closed on 30.05.2018	City	Public exposure assessment	Automatic: PM10<br>Manual: PM10, BaP(PM10)

"Station sites" are defined in the Polish law in reference to the types of specific pollutions dominating in selected locations. These determine the types of monitoring stations, based on the selection of the pollutants being measured (last column).

Table S2. Ranges of the 1h concentrations in the Polish air quality index (AQI) for selected pollutants [3].

Air quality index	SO_2 [μg/m³]	NO_2 [μg/m³]	CO [μg/m³]	PM_{10} [μg/m³]	$PM_{2.5}$ [μg/m³]	O_3 [μg/m³]	C_6H_6 [μg/m³]
Very good	0–50	0–40	0–3,000	0–20	0–13	0–70	0–6
Good	50.1–100	40.1–100	3,001–7,000	20.1–50	13.1–35	70.1–120	6.1–11
Moderate	100.1–200	100.1–150	7,001–11,000	50.1–80	35.1–55	120.1–150	11.1–16
Average	200.1–350	150.1–200	11,001–15,000	80.1–110	55.1–75	150.1–180	16.1–21
Bad	350.1–500	200.1–400	15,001–21,000	110.1–150	75.1–110	180.1–240	21.1–51
Very bad	>500	>400	>21,000	>150	>110	>240	>51

Table S3. The Polish AQI index and the corresponding health recommendations for residents [3].

Air quality index	Health recommendations
Very good	Good air quality. Air pollution is not a threat. Ideal conditions for outdoor activities.
Good	Good air quality. Air pollution poses a minimum threat to those at risk. Very good conditions for outdoor activities.
Moderate	Acceptable air quality. Air pollution can pose a risk to people at risk. Good conditions for outdoor activities.
Average	Average air quality. Air pollution is a threat to those at risk who may experience health effects. Other people should limit spending time outdoors, especially if they experience such symptoms as coughing or sore throat.
Bad	Bad air quality. People at risk should avoid going outside. Other residents should limit staying outdoors. Outdoor activities are not recommended.
Very bad	Dangerously bad air quality. People at risk should definitely avoid going outside. Others should keep exits to a minimum. All outdoor activities are discouraged.

Table S4. Description of the enrichment indices used in the study [4].

Enrichment factors	Formulas	Explanations	Limit values	Classification	References
Geo-accumulation index I_{geo}	$I_{geo}=log_2*(C_i/1.5*B_n)$	C_i – element content in PM; B_n – background value; 1.5 – constant	$I_{geo} \leq 0$	Class 0 – practically uncontaminated	[5-7]
			$0 \leq I_{geo} < 1$	Class 1 – uncontaminated to moderately contaminated	
			$1 \leq I_{geo} < 2$	Class 2 – moderately contaminated	
			$2 \leq I_{geo} < 3$	Class 3 – moderately to heavily contaminated	
			$3 \leq I_{geo} < 4$	Class 4 – heavily contaminated	
			$4 \leq I_{geo} < 5$	Class 5 – heavily to extremely contaminated	
			$5 \leq I_{geo}$	Class 6 – extremely contaminated	
Contamination factor CF	$CF = Cm_i/C_{ref}$	Cm_i – mean element concentration in PM; C_{ref} – reference value of element	CF <1	low contamination	[8,9]
			1≤ CF <3	moderate contamination	
			3≤ CF <6	considerable contamination	
			6 ≤CF	very high contamination	
Enrichment factor EF	$EF=(C_i/C_{ref})/(B_i/B_{ref})$	C_i – element content in PM; C_{ref} – Fe content in sample; B_i – reference content of a single element; B_{ref} – Fe reference content	EF ≤1	no enrichment	[10,11]
			1< EF ≤3	minor enrichment	
			3< EF ≤5	moderate enrichment	
			5< EF ≤10	moderately severe enrichment	
			10< EF ≤25	severe enrichment	
			25< EF ≤50	very severe enrichment	
			EF >50	extremely severe enrichment	
Ecological risk index ERI	$ERI = Tr_i \times CF_i$	Tr – toxicity response coefficient of a single element; CF_i – contamination factor of a single element	ERI <40	low potential ecological risk	[8, 12-15]
			40≤ ERI <80	moderate potential ecological risk	
			80 ≤ERI <160	considerable potential ecological risk	
			160≤ ERI <320	high potential ecological risk	
			320≤ ERI	very high potential ecological risk	

Modified hazard quotient mHQ	$mHQ = [Ci\,(\frac{1}{TELi} + \frac{1}{PELi} + \frac{1}{SELi})]^{2}$	Ci – element concentration in PM; TEL – threshold effect level; PEL – probable effect level; SEL – severe effect level for a single element	mHQ <0.5<br>0.5< mHQ <1.0<br>1.0< mHQ <1.5<br>1.5< mHQ <2.0<br>2.0< mHQ <2.5<br>2.5< mHQ <3.0<br>3.0< mHQ <3.5<br>mHQ >3.5	nil to very low severity of contamination<br>very low severity of contamination<br>low severity of contamination<br>moderate severity of contamination<br>considerable severity of contamination<br>high severity of contamination<br>very high severity of contamination<br>extreme severity of contamination	[16,17]

Table S5. Exposure parameters used for the risk assessment calculations under resident scenario in the study.

Exposure parameters	Adult	Child	References
IR_{ing} – PM ingestion rate (mg/kg)	100	200	[18]
IR_{inh} – inhalation rate for person (m^3/h)	0.83	0.31	[19,20]
CF – unit conversion factor (kg/mg)	10^{-6}	10^{-6}	[18]
FI – fraction ingested from a contaminated source (unitless)	1	1	[21]
ET – exposure time for person (h/day)	24	24	site specific*
ED – exposure duration (years)	24	6	[21,22]
EF – exposure frequency (days/year)	350	350	site specific*
EV – event frequency (events/day)	1	1	site specific*
AF – soil-to-skin adherence factor (mg/cm^2-event)	0.07	0.2	[18]
SA – skin surface area available for contact (cm^2)	6,032	2,373	[23]
PEF – soil-to-air particulate emission factor (m^3/kg)	1.36×10^9	1.36×10^9	[19]
BW – body weight (kg)	70	15	[18]
AT – averaging time – non-carcinogens (days)	8,760	2,190	[21]
AT – averaging time – carcinogens (days)	25,550	25,550	[21]
AT – averaging time – non-carcinogens (hours)	210,240	52,560	[24]
AT – averaging time – carcinogens (hours)	613,200	613,200	[24]

* site specific; assumption: two weeks of holidays each year

Table S6. Toxicological parameters used for the risk assessment calculations under resident scenario in the study.

Toxicological parameters	RfC (1) mg/m³	IUR (1) (mg/m³)-1	RfD (1) mg/kg-day	SF (1) (mg/kg-day)-1	RBA (1) unitless	ABSd (1) unitless
**Ambient air**						
SO_2	-	-	-	-	-	-
NO_2	-	-	1.10×10^{-2}	-	-	-
CO	-	-	-	-	-	-
Benzen	3.00×10^{-2}	7.80×10^{-9}	4.00×10^{-3}	5.50×10^{-2}	-	-
PM2.5	5.00×10^{-3} (2)	-	-	-	-	-
PM10	-	-	1.10×10^{-2}	-	-	-
Pb(PM10)	-	-	3.50×10^{-3} (3)	4.20×10^{-2} (4)	1	-
As(PM10)	1.50×10^{-5}	4.30×10^{-6}	3.00×10^{-4}	1.50×10^{0}	0.6	0.03
Cd(PM10)	1.00×10^{-5}	1.80×10^{-6}	1.00×10^{-3}	-	1	0.001
Ni(PM10)	9.00×10^{-5}	2.60×10^{-10}	2.00×10^{-2}	-	1	0.01
BaP(PM10)	2.00×10^{-6}	6.00×10^{-7}	3.00×10^{-4}	1.00×10^{0}	-	0.13
BaA(PM10)	-	6.00×10^{-8}	-	1.00×10^{-1}	-	0.13
BbF (PM10)	-	6.00×10^{-8}	-	1.00×10^{-1}	-	0.13
BjF(PM10)	-	1.10×10^{-7}	-	1.20×10^{0}	-	0.13
BkF(PM10)	-	6.00×10^{-9}	-	1.00×10^{-2}	-	0.13
DBahA(PM10)	-	6.00×10^{-7}	-	1.00×10^{0}	-	0.13
**Deposited PM**						
Al	5.00×10^{-3}	-	1.00×10^{0}	-	1	-
As	1.50×10^{-5}	4.30×10^{-6}	3.00×10^{-4}	1.50×10^{0}	0.6	0.03
Ba	5.00×10^{-4}	-	2.00×10^{-1}	-	1	-
Be	2.00×10^{-5}	2.40×10^{-6}	2.00×10^{-3}	-	1	-
Cd	1.00×10^{-5}	1.80×10^{-6}	1.00×10^{-3}	-	1	0.001
Co	6.00×10^{-6}	9.00×10^{-6}	3.00×10^{-4}	-	1	-
Cr(III)	-	-	1.50×10^{0}	-	1	-
Cr(VI)	1.00×10^{-4}	8.40×10^{-5}	3.00×10^{-3}	5.00×10^{-1}	1	-
Cu	-	-	4.00×10^{-2}	-	1	-
Fe	-	-	7.00×10^{-1}	-	1	-
Li	-	-	2.00×10^{-3}	-	1	-
Mn	5.00×10^{-5}	-	2.40×10^{-2}	-	1	-
Ni	9.00×10^{-5}	2.60×10^{-10}	2.00×10^{-2}	-	1	0.01
Pb	-	-	3.50×10^{-3} (3)	4.20×10^{-2} (4)	1	-
Sn	-	-	6.00×10^{-1}	-	1	-
Sr	-	-	6.00×10^{-1}	-	1	-
V	1.00×10^{-4}	-	5.00×10^{-3}	-	1	-
Zn	-	-	3.00×10^{-1}	-	1	-
Zr	-	-	8.00×10^{-5}	-	1	-

RfC, reference concentration; RfD, reference dose; IUR, inhalation unit risk; SF, slope factor; RBA, relative bioavailability factor; ABS_d, dermal absorption factor

- not available

[1][25]; [2][26]; [3][27]; [4][28]

Table S7. Changes in annual air pollutant contents in Kraków in 2005-2020 [3], with permissible levels.

Year	NO_2 $\mu g/m^3$	SO_2 $\mu g/m^3$	CO $\mu g/m^3$	C_6H_6 $\mu g/m^3$	O_3 $\mu g/m^3$	PM10 $\mu g/m^3$	PM2.5 $\mu g/m^3$	As (PM10) ng/m^3	Cd (PM10) ng/m^3	Ni (PM10) ng/m^3	Pb (PM10) ng/m^3	BaP (PM10) ng/m^3	BaA (PM10) ng/m^3	BbF (PM10) ng/m^3	BjF (PM10) ng/m^3	BkF (PM10) ng/m^3	DBahA (PM10) ng/m^3
2005	**63.0**	-	-	**5.5**	-	**73.0**	-	-	-	-	-	-	-	-	-	-	-
2006	**66.0**	-	-	**5.9**	-	**86.5**	-	-	-	-	-	-	-	-	-	-	-
2007	**45.5**	10.0	1004	4.5	-	**69.5**	**59.0**	-	-	-	-	-	-	-	-	-	-
2008	**48.0**	8.0	685	3.7	-	**70.5**	**38.0**	-	-	-	-	-	-	-	-	-	-
2009	**50.5**	9.0	977	4.0	-	**70.5**	**43.0**	-	-	-	-	-	-	-	-	-	-
2010	**45.3**	8.5	997	4.0	34	**63.3**	**45.7**	-	-	-	-	-	-	-	-	-	-
2011	**44.7**	8.5	838	-	34	**65.3**	**45.7**	-	-	-	-	-	-	-	-	-	-
2012	**44.7**	10.5	851	-	34	**58.0**	**39.3**	-	-	-	-	-	-	-	-	-	-
2013	**44.7**	8.5	833	3.0	35	**51.3**	**36.7**	-	-	-	-	-	-	-	-	-	-
2014	38.0	7.0	881	2.8	33	**52.7**	**36.0**	1.37	0.92	1.89	0.027	6.9	3.0	4.2	4.5	2.7	1.1
2015	**41.0**	7.0	769	2.8	38	**54.3**	**36.3**	2.09	1.19	1.91	0.033	7.8	30.	3.8	4.1	3.4	2.3
2016	**41.3**	6.0	701	2.1	34	**44.0**	**32.0**	1.26	0.69	1.44	0.133	4.2	6.8	3.0	2.2	3.7	0.7
2017	**41.5**	7.0	753	2.2	38	**44.0**	**33.0**	1.26	0.57	1.53	0.019	4.7	7.8	3.4	2.7	2.3	0.6
2018	**40.3**	6.0	693	2.3	41	**42.9**	**31.7**	0.98	0.52	1.90	0.017	5.3	6.0	2.5	1.8	1.9	0.4
2019	38.8	5.0	602	1.3	40	34.8	25.0	0.88	0.46	1.61	0.013	4.5	4.4	2.3	1.7	1.6	0.3
2020	35.3*	5.3*	557*	0.8*	45*	34.4*	26.1*	1.27*	0.42*	1.33*	0.016*	8.5*	9.9*	4.4*	3.6*	3.2*	0.7*
average	45.5	7.6	796	3.2	35	57.2	37.7	1.30	0.68	1.66	0.037	5.9	5.8	3.4	2.9	2.7	0.9
annual permissible level [29]	40	125	10,000	5	-	40	25 (20**)	6***	5***	20***	500	-	-	-	-	-	-

- data not available; * until the end of May 2020; ** since 2020; *** [30]; bold: the concentrations exceeding the recommended levels.

Table S8. Changes in monthly air pollutant contents in 2018 in Kraków; average values of all the monitoring stations [3].

**Month.**	**NO_2.** µg/m³	**SO_2** µg/m³	**CO** µg/m³	**C_6H_6** µg/m³	**PM10** µg/m³	**PM2.5** µg/m³	**O_3** µg/m³	**As (PM10)** ng/m³	**Cd (PM10)** ng/m³	**Ni (PM10)** ng/m³	**Pb (PM10)** ng/m³	**BaP (PM10)** ng/m³	**BaA (PM10)** ng/m³	**BbF (PM10)** ng/m³	**BjF (PM10)** ng/m³	**BkF (PM10)** ng/m³	**DBahA (PM10)** ng/m³
January	40	8.0	1009	4.9	56	47	24	2.03	0.59	1.59	0.025	12.1	12.1	3.79	2.83	3.57	0.67
February	39	7.9	803	3.5	61	53	33	1.51	0.64	1.91	0.021	12.7	17.2	5.57	4.45	4.48	0.98
March	47	11.3	822	3.9	70	51	41	1.45	0.53	1.69	0.019	10.7	15.1	5.58	4.62	4.32	1.09
April	45	4.9	567	1.6	41	23	48	0.57	0.39	2.46	0.012	1.77	1.55	1.14	0.67	0.73	0.18
May	38	5.1	501	1.1	30	19	65	0.50	0.40	2.60	0.014	0.61	0.30	0.37	0.18	0.20	0.05
June	38	4.6	482	0.8	26	17	62	0.50	0.28	1.75	0.009	0.22	0.24	0.31	0.14	0.16	0.03
July	38	4.7	482	0.8	25	16	56	0.50	0.32	1.08	0.008	0.19	0.20	0.29	0.14	0.15	0.02
August	41	4.9	548	1.2	29	19	54	0.53	0.38	2.10	0.012	0.55	0.22	0.29	0.15	0.15	0.02
September	41	4.4	596	1.2	31	22	43	0.52	0.41	1.53	0.013	0.76	0.66	0.77	0.39	0.42	0.06
October	43	4.6	762	2.3	48	35	30	0.82	0.57	1.52	0.019	3.73	2.34	2.35	1.45	1.43	0.28
November	37	5.5	939	3.6	55	46	18	1.19	0.82	1.99	0.024	8.70	8.95	5.02	3.12	3.38	0.70
December	39	7.2	809	3.0	42	34	20	1.12	0.58	1.47	0.016	10.6	12.7	5.04	3.79	3.70	0.80

Table S9. Changes in daily air pollutant contents in a selected winter month, average values for Kraków, March 2018 [3], with recommended concentrations.

Day	NO_2 [μg/m³]	SO_2 [μg/m³]	CO [μg/m³]	C_6H_6 [μg/m³]	O_3 [μg/m³]	O_3 8-hour [μg/m³]	PM10 [μg/m³]	PM2.5 [μg/m³]
1 March	50	14	888	5.5	37	66	**104**	**81**
2 March	40	16	825	4.9	62	86	**99**	**76**
3 March	55	18	855	5.2	48	87	**104**	**80**
4 March	60	19	1399	7.8	46	86	**152**	**115**
5 March	81	21	1467	9.0	37	67	**150**	**111**
6 March	70	17	1339	7.5	19	48	**100**	**84**
7 March	61	12	1262	7.0	22	52	**92**	**72**
8 March	56	10	1049	3.9	25	58	**67**	**52**
9 March	57	11	1110	5.0	28	70	**76**	**58**
10 March	56	11	1245	6.3	27	67	**95**	**63**
11 March	45	9	1111	4.7	43	82	**74**	**48**
12 March	39	9	602	1.6	58	69	39	19
13 March	45	8	652	1.5	43	63	34	17
14 March	47	8	797	3.0	10	19	49	**40**
15 March	34	7	493	2.6	23	40	43	**31**
16 March	31	6	615	3.0	22	36	38	**33**
17 March	16	5	244	0.9	74	80	22	16
18 March	13	9	241	0.8	82	85	30	17
19 March	27	7	414	1.4	81	85	32	23
20 March	45	10	558	2.5	50	78	48	**38**
21 March	45	14	598	2.7	45	80	**56**	**39**
22 March	58	12	769	3.6	37	79	**75**	**40**
23 March	57	14	1156	5.9	29	62	**103**	**75**
24 March	48	14	916	5.0	55	92	**86**	**67**
25 March	50	15	791	3.9	42	93	**76**	**58**
26 March	58	12	827	3.6	31	67	**77**	**53**
27 March	45	9	688	2.9	26	44	**64**	**48**
28 March	40	7	555	2.3	45	71	**51**	**36**
29 March	53	10	756	3.1	23	51	**59**	**41**
30 March	44	8	604	2.6	45	88	43	**31**
31 March	34	9	688	3.2	43	64	34	25
24h permissible level [29]	-	125	-	-	-	120*	50	25**

* 8h permissible level

** guideline value [31]

bold: the concentrations exceeding the recommended levels

2 March weekend marking

Table S10. Changes in daily air pollutant contents in a selected summer month, average values for Kraków, July 2018 [3], with recommended concentrations.

Day	NO_2 [µg/m³]	SO_2 [µg/m³]	CO [µg/m³]	C_6H_6 [µg/m³]	O_3 [µg/m³]	O_3 8-hour [µg/m³]	PM10 [µg/m³]	PM2.5 [µg/m³]
1 July	21	3.3	360	0.4	48	55	12	6
2 July	31	3.8	433	0.5	47	77	18	9
3 July	44	5.1	464	0.8	52	107	24	13
4 July	57	6.3	524	1.0	69	**142**	29	16
5 July	65	5.1	613	1.1	79	**156**	34	19
6 July	54	4.5	595	0.9	76	**123**	32	23
7 July	32	4.5	444	0.5	65	105	25	15
8 July	30	4.4	398	0.6	64	102	23	13
9 July	50	4.7	548	1.0	48	102	30	19
10 July	45	3.8	487	0.9	58	117	29	18
11 July	41	4.6	601	1.3	30	59	28	20
12 July	34	4.5	442	0.6	59	92	19	12
13 July	31	3.6	425	0.5	65	101	16	10
14 July	24	4.1	381	0.4	66	86	20	12
15 July	22	4.5	378	0.5	64	85	19	12
16 July	39	5.0	524	0.7	44	70	24	16
17 July	30	4.1	423	0.5	45	52	17	14
18 July	27	3.8	417	0.5	39	53	13	9
19 July	26	3.9	415	0.5	35	41	20	17
20 July	33	4.3	515	0.8	35	68	31	22
21 July	46	5.1	528	1.0	70	**137**	35	25
22 July	37	6.1	512	1.6	78	**144**	34	27
23 July	42	4.8	430	0.7	60	111	32	18
24 July	45	4.0	399	0.6	77	130	34	19
25 July	39	4.4	455	0.8	54	99	30	17
26 July	37	4.6	480	0.8	51	78	21	12
27 July	36	6.7	503	0.9	67	98	24	14
28 July	37	6.1	571	1.0	54	117	26	14
29 July	29	4.6	485	0.6	63	106	23	14
30 July	43	4.0	584	1.3	30	69	24	15
31 July	33	6.9	607	1.7	58	93	29	15
24h permissible level [29]	-	125	-	-	-	120*	50	25**

* 8h permissible level

** guideline value [31]

bold: the concentrations exceeding the recommended levels

1 July weekend marking

Table S11. Daily and hourly SO_2 content changes, with the hourly Polish AQI index, average values for Kraków, March 2018 [3] (weekend marking in grey).

SO_2																															
**Day / Hour**	**1**	**2**	**3**	**4**	**5**	**6**	**7**	**8**	**9**	**10**	**11**	**12**	**13**	**14**	**15**	**16**	**17**	**18**	**19**	**20**	**21**	**22**	**23**	**24**	**25**	**26**	**27**	**28**	**29**	**30**	**31**
01:00	12	14	23	19	14	29	9	10	8	9	10	8	7	10	8	6	4	9	7	7	21	18	12	9	11	8	15	4	11	12	6
02:00	9	13	21	19	15	30	7	7	7	9	10	10	6	8	9	5	4	6	7	7	20	15	9	8	10	7	14	4	13	9	6
03:00	11	11	18	20	20	26	6	6	7	7	8	9	6	7	9	6	3	8	6	6	21	13	8	8	10	7	15	4	9	8	7
04:00	12	11	16	20	18	24	6	6	6	6	7	8	6	7	9	5	4	10	8	7	25	11	8	7	12	8	15	4	8	10	7
05:00	12	11	11	17	18	22	8	5	6	7	7	10	5	7	7	5	4	12	6	7	27	10	8	7	12	9	11	4	6	7	8
06:00	12	12	11	18	16	19	9	8	8	7	7	11	6	7	6	6	4	12	5	8	26	10	8	8	10	10	11	5	5	7	8
07:00	12	12	11	14	16	20	9	10	10	8	7	12	7	9	6	5	3	9	5	10	22	14	11	9	10	12	13	5	5	9	8
08:00	16	14	18	23	26	23	9	13	11	9	9	13	8	11	6	6	3	10	8	11	16	17	23	15	22	16	15	6	7	15	8
09:00	22	25	25	33	32	23	10	18	9	15	18	12	11	12	6	7	3	14	8	11	9	12	20	20	33	17	15	7	8	16	9
10:00	28	25	26	28	32	23	16	20	11	18	16	13	9	11	6	9	4	15	8	10	5	9	17	24	30	19	11	9	14	13	9
11:00	17	21	22	22	37	23	26	17	14	15	14	13	8	10	8	7	4	15	10	10	4	11	16	19	23	16	8	7	11	10	10
12:00	16	21	21	24	23	25	19	11	16	13	11	12	9	10	9	6	4	15	10	10	4	12	23	20	17	13	6	9	10	7	16
13:00	16	19	18	17	22	17	18	9	10	12	10	7	8	8	9	7	6	12	12	13	4	13	23	20	14	11	6	8	10	5	22
14:00	15	19	17	16	23	16	14	7	8	10	8	6	7	7	8	7	5	9	10	12	5	11	19	17	13	9	7	8	10	4	18
15:00	14	15	15	16	19	13	13	7	8	9	7	6	7	7	11	7	5	8	10	9	13	10	18	15	13	7	9	7	12	5	17
16:00	11	15	15	15	15	11	12	7	8	9	7	6	7	7	7	7	6	5	8	7	13	10	15	13	12	8	9	8	15	5	11
17:00	9	12	14	19	18	11	11	8	7	9	7	6	7	7	6	7	4	5	7	6	9	10	12	11	13	10	6	7	12	9	9
18:00	9	14	14	17	23	11	13	9	7	11	8	8	8	7	7	5	4	5	6	6	7	9	11	10	14	12	5	7	10	9	8
19:00	10	14	14	19	21	10	12	11	13	12	7	7	9	7	8	5	8	5	6	6	7	9	11	10	10	13	4	8	8	9	6
20:00	11	11	16	16	20	9	11	11	18	13	8	7	10	6	8	5	8	4	7	8	7	11	12	15	12	14	4	8	9	8	6
21:00	14	16	17	18	19	8	12	11	17	12	7	7	10	7	7	5	7	8	8	11	10	12	12	19	14	15	4	8	10	7	6
22:00	16	20	19	19	18	8	14	10	15	11	7	7	11	8	6	4	5	9	7	15	15	15	11	21	14	18	3	8	10	6	7
23:00	16	22	18	18	20	8	13	10	15	11	7	6	11	8	6	4	7	9	6	17	18	13	10	19	14	18	3	9	11	6	7
24:00	14	24	19	17	22	10	12	8	14	12	7	6	11	8	6	4	10	7	7	19	17	13	10	14	10	18	3	10	11	7	6

Colors refer to the following air quality index (AQI) codes: dark green – very good; green – good; yellow – moderate; orange – average; red – bad; maroon – very bad.

Table S12. Daily and hourly CO content changes, with the hourly Polish AQI index, average values for Kraków, March 2018 [3] (weekend marking in grey).

CO																															
**Day / Hour**	**1**	**2**	**3**	**4**	**5**	**6**	**7**	**8**	**9**	**10**	**11**	**12**	**13**	**14**	**15**	**16**	**17**	**18**	**19**	**20**	**21**	**22**	**23**	**24**	**25**	**26**	**27**	**28**	**29**	**30**	**31**
01:00	693	761	524	1937	1653	1357	1522	1141	1614	1706	1769	746	457	981	535	738	218	202	225	365	797	811	1820	1366	964	722	799	377	541	722	720
02:00	807	967	556	2071	1695	1295	1533	1257	1659	2070	1727	833	429	976	507	645	189	173	247	358	787	719	1872	1484	946	810	754	310	639	817	637
03:00	1004	872	606	2113	1752	1470	1402	1060	1630	1752	1747	765	391	942	601	581	166	167	267	365	855	670	1940	1505	962	808	787	323	479	794	841
04:00	1200	1015	650	2255	1721	1509	1402	1144	1633	1667	1703	539	408	944	523	533	137	203	246	359	897	618	1854	1517	1021	874	804	335	535	717	998
05:00	1240	1015	736	2316	1854	1526	1481	1259	1699	1675	1703	509	447	947	354	600	119	137	241	324	970	588	1801	1493	1398	1032	874	533	500	633	1008
06:00	1356	1116	809	2215	1793	1518	1811	1528	1790	1732	1718	567	602	1002	412	545	128	111	294	604	1093	645	1790	1450	1297	1090	1043	779	649	1001	974
07:00	1488	1277	907	2120	1891	1512	1754	1864	1911	1774	1745	646	858	1287	691	584	156	126	367	663	744	794	1750	1285	1268	1275	1181	897	663	1011	911
08:00	1880	1147	954	1976	2016	1467	1918	1958	1943	1907	1676	894	1110	1264	502	633	162	166	391	671	381	705	1706	1125	1215	1308	1191	748	886	801	872
09:00	1344	1281	855	1665	2170	1177	2126	1306	1067	1690	1255	958	689	1181	495	812	229	195	355	477	311	580	1295	1164	959	1093	1157	629	1023	539	880
10:00	844	1036	724	1293	1771	1348	2059	928	671	1070	748	673	555	813	432	880	253	161	455	519	295	469	812	753	684	813	934	560	861	371	579
11:00	525	862	607	758	1005	1388	1707	609	629	675	707	642	523	686	404	699	289	190	436	491	291	315	577	632	562	605	707	519	706	312	778
12:00	569	792	555	721	1022	856	1149	575	456	642	559	535	464	720	373	691	284	221	407	541	281	318	570	603	529	550	631	400	474	292	654
13:00	526	821	552	587	991	822	573	476	407	564	528	378	455	595	484	800	276	206	432	567	300	329	575	522	518	526	689	361	466	282	588
14:00	551	791	558	592	1002	911	581	478	385	518	535	393	480	439	351	842	288	221	411	529	264	323	599	441	480	527	518	398	545	337	525
15:00	590	787	541	610	1105	1375	635	510	434	566	532	415	496	428	366	967	307	300	515	512	328	391	554	426	509	495	543	499	573	353	431
16:00	605	656	541	644	1015	1384	703	542	405	572	541	451	595	675	391	838	269	297	561	498	337	421	552	416	579	526	452	484	587	394	291
17:00	532	586	514	788	981	1273	722	594	411	711	495	499	619	794	371	724	306	315	528	566	368	423	561	433	582	702	425	529	613	440	352
18:00	633	620	671	904	1300	1628	801	657	562	1000	642	582	670	654	426	503	369	353	637	570	434	599	655	485	737	751	427	681	715	498	454
19:00	694	651	784	1055	1329	1225	940	752	678	901	806	486	690	643	467	444	312	321	524	674	419	810	717	528	710	846	552	711	891	567	501
20:00	879	530	1076	1159	1476	1259	1083	809	984	1060	1049	540	748	674	690	385	316	399	557	756	546	1234	921	731	665	893	531	652	1068	747	559
21:00	799	572	1372	1408	1360	1595	1117	1149	1053	1371	1297	614	829	620	859	347	290	376	512	756	797	1448	1025	765	767	1054	495	626	1366	736	552
22:00	826	533	1743	1307	1447	1695	1121	1372	1270	1344	1296	554	984	611	624	355	291	273	496	787	980	1610	1021	980	631	938	396	761	1388	780	737
23:00	907	567	1800	1497	1501	1552	1092	1551	1620	1573	1126	617	1108	669	563	339	241	398	439	826	960	1734	1410	1019	687	823	305	676	1194	788	863
24:00	816	555	1891	1568	1490	1373	1063	1657	1725	1772	762	619	1042	575	586	276	271	264	388	728	924	1904	1364	854	716	782	317	538	775	685	803

Colors refer to the following air quality index (AQI) codes: dark green – very good; green – good; yellow – moderate; orange – average; red – bad; maroon – very bad.

Table S13. Changes in monthly air-pollutant contents in the first half of 2020 in Kraków, average values from all the monitoring stations [3].

Month	NO_2 µg/m³	SO_2 µg/m³	CO µg/m³	C_6H_6 µg/m³	PM10 µg/m³	PM2.5 µg/m³	O_3 µg/m³	As (PM10) ng/m³	Cd (PM10) ng/m³	Ni (PM10) ng/m³	Pb (PM10) ng/m³	BaP (PM10) ng/m³	BaA (PM10) ng/m³	BbF (PM10) ng/m³	BjF (PM10) ng/m³	BkF (PM10) ng/m³	DBahA (PM10) ng/m³
January	43	7.75	842	1.57	53	47	21	1.66	0.55	1.74	0.022	11.6	14.5	6.30	5.31	4.48	1.02
February	39	5.05	563	0.70	27	21	41	0.87	0.28	0.91	0.01	5.29	5.27	2.43	1.92	1.75	0.37
March	35	5.25	528	0.70	37	28	49	-	-	-	-	5.73	-	-	-	-	-
April	32	5.00	407	0.47	33	22	62	-	-	-	-	-	-	-	-	-	-
May	28	3.60	376	0.40	21	14	51	-	-	-	-	-	-	-	-	-	-

- data not available

Table S14. Estimated daily intake values for the resident of Kraków, in reference to exposure pathways.

Pollutant	Inhalation				Ingestion				Dermal contact			
	$EC_{non\text{-}carc}$ (mg/m^3)		EC_{carc} (mg/m^3)		$ADD_{ing\ non\text{-}canc}$ (mg/kg-day)		$ADD_{ing\ canc}$ (mg/kg-day)		$ADD_{der\ non\text{-}canc}$ (mg/kg-day)		$ADD_{der\ canc}$ (mg/kg-day)	
	adult	child	adult	child	adult	child	adult	child	adult	child	adult	child
**Ambient air**												
SO_2	7.29×10^{-3}	7.29×10^{-3}	2.50×10^{-3}	6.25×10^{-4}	–				–			
NO_2	4.36×10^{-2}	4.36×10^{-2}	1.50×10^{-2}	3.74×10^{-3}								
CO	7.63×10^{-1}	7.63×10^{-1}	2.62×10^{-1}	6.54×10^{-2}								
Benzen	3.07×10^{-3}	3.07×10^{-3}	1.05×10^{-3}	2.63×10^{-4}								
PM2.5	3.62×10^{-2}	3.62×10^{-2}	1.24×10^{-2}	3.10×10^{-3}								
PM10	5.48×10^{-2}	5.48×10^{-2}	1.88×10^{-2}	4.70×10^{-3}								
Pb (PM10)	3.55×10^{-5}	3.55×10^{-5}	1.22×10^{-5}	3.04×10^{-6}								
As (PM10)	1.25×10^{-6}	1.25×10^{-6}	4.27×10^{-7}	1.07×10^{-7}								
Cd (PM10)	6.52×10^{-7}	6.52×10^{-7}	2.24×10^{-7}	5.59×10^{-8}								
Ni (PM10)	1.59×10^{-6}	1.59×10^{-6}	5.46×10^{-7}	1.36×10^{-7}								
BaP (PM10)	5.66×10^{-6}	5.66×10^{-6}	1.94×10^{-6}	4.85×10^{-7}								
BaA (PM10)	5.60×10^{-6}	5.60×10^{-6}	1.92×10^{-6}	4.80×10^{-7}								
BbF (PM10)	3.23×10^{-6}	3.23×10^{-6}	1.11×10^{-6}	2.77×10^{-7}								
BjF (PM10)	2.82×10^{-6}	2.82×10^{-6}	9.67×10^{-7}	2.42×10^{-7}								
BkF (PM10)	2.58×10^{-6}	2.58×10^{-6}	8.84×10^{-7}	2.21×10^{-7}								
DBahA (PM10)	8.34×10^{-7}	8.34×10^{-7}	2.86×10^{-7}	7.15×10^{-8}								
	$ADD_{inh\ non\text{-}canc}$ (mg/kg-day)		$ADD_{inh\ canc}$ (mg/kg-day)		$ADD_{ing\ non\text{-}canc}$ (mg/kg-day)		$ADD_{ing\ canc}$ (mg/kg-day)		$ADD_{der\ non\text{-}canc}$ (mg/kg-day)		$ADD_{der\ canc}$ (mg/kg-day)	
	adult	child	adult	adult	adult	child	adult	child	adult	child	adult	child
SO_2	2.08×10^{-3}	4.86×10^{-3}	7.14×10^{-4}	4.16×10^{-4}	–				–			
NO_2	1.25×10^{-2}	2.91×10^{-2}	4.27×10^{-3}	2.49×10^{-3}								
CO	2.18×10^{-1}	5.09×10^{-1}	7.47×10^{-2}	4.36×10^{-2}								
Benzen	8.77×10^{-4}	2.05×10^{-3}	3.01×10^{-4}	1.75×10^{-4}								

PM2.5	1.03×10^{-2}	2.41×10^{-2}	3.54×10^{-3}	2.07×10^{-3}
PM10	1.57×10^{-2}	3.66×10^{-2}	5.37×10^{-3}	3.13×10^{-3}
Pb (PM10)	1.01×10^{-5}	2.37×10^{-5}	3.48×10^{-6}	2.03×10^{-6}
As (PM10)	3.56×10^{-7}	8.31×10^{-7}	1.22×10^{-7}	7.12×10^{-8}
Cd (PM10)	1.86×10^{-7}	4.35×10^{-7}	6.39×10^{-8}	3.73×10^{-8}
Ni (PM10)	4.55×10^{-7}	1.06×10^{-6}	1.56×10^{-7}	9.10×10^{-8}
BaP (PM10)	1.62×10^{-6}	3.77×10^{-6}	5.54×10^{-7}	3.23×10^{-7}
BaA (PM10)	1.60×10^{-6}	3.73×10^{-6}	5.49×10^{-7}	3.20×10^{-7}
BbF (PM10)	9.23×10^{-7}	2.15×10^{-6}	3.17×10^{-7}	1.85×10^{-7}
BjF (PM10)	8.05×10^{-7}	1.88×10^{-6}	2.76×10^{-7}	1.61×10^{-7}
BkF (PM10)	7.37×10^{-7}	1.72×10^{-6}	2.53×10^{-7}	1.47×10^{-7}
DBahA (PM10)	2.38×10^{-7}	5.56×10^{-7}	8.17×10^{-8}	4.77×10^{-8}

Deposited PM

	$EC_{non-carc}$ (mg/m³)		EC_{carc} (mg/m³)		$ADD_{ing\ non-canc}$ (mg/kg-day)		$ADD_{ing\ canc}$ (mg/kg-day)		$ADD_{der\ non-canc}$ (mg/kg-day)		$ADD_{der\ canc}$ (mg/kg-day)	
	adult	child	adult	child	adult	child	adult	child	adult	child	adult	child
Al	1.07×10^{-4}	1.07×10^{-4}	3.84×10^{-5}	9.21×10^{-6}								
As	2.48×10^{-7}	2.48×10^{-7}	8.86×10^{-8}	2.12×10^{-8}								
Ba	1.89×10^{-6}	1.89×10^{-6}	6.75×10^{-7}	1.62×10^{-7}								
Be	9.14×10^{-9}	9.14×10^{-9}	3.27×10^{-9}	7.83×10^{-10}								
Cd	1.95×10^{-8}	1.95×10^{-8}	6.96×10^{-9}	1.67×10^{-9}								
Co	3.98×10^{-8}	3.98×10^{-8}	1.42×10^{-8}	3.41×10^{-9}								
Cr(III)	1.52×10^{-6}	1.52×10^{-6}	5.45×10^{-7}	1.31×10^{-7}	–				–			
Cr(VI)	1.52×10^{-6}	1.52×10^{-6}	5.45×10^{-7}	1.31×10^{-7}								
Cu	2.11×10^{-6}	2.11×10^{-6}	7.53×10^{-7}	1.81×10^{-7}								
Fe	6.84×10^{-4}	6.84×10^{-4}	2.45×10^{-4}	5.87×10^{-5}								
Li	3.57×10^{-7}	3.57×10^{-7}	1.28×10^{-7}	3.06×10^{-8}								
Mn	1.06×10^{-5}	1.06×10^{-5}	3.77×10^{-6}	9.04×10^{-7}								
Ni	4.14×10^{-7}	4.14×10^{-7}	1.48×10^{-7}	3.55×10^{-8}								

Pb	1.45×10^{-6}	1.45×10^{-6}	5.17×10^{-7}	1.24×10^{-7}
Sn	2.12×10^{-7}	2.12×10^{-7}	7.56×10^{-8}	1.81×10^{-8}
Sr	2.38×10^{-6}	2.38×10^{-6}	8.50×10^{-7}	2.04×10^{-7}
V	5.08×10^{-7}	5.08×10^{-7}	1.82×10^{-7}	4.35×10^{-8}
Zn	9.85×10^{-5}	9.85×10^{-5}	3.52×10^{-5}	8.44×10^{-6}
Zr	7.70×10^{-8}	7.70×10^{-8}	2.75×10^{-8}	6.60×10^{-9}

	**$ADD_{inh\ non\text{-}canc}$ (mg/kg-day)**		**$ADD_{inh\ canc}$ (mg/kg-day)**		**$ADD_{ing\ non\text{-}canc}$ (mg/kg-day)**		**$ADD_{ing\ canc}$ (mg/kg-day)**		**$ADD_{derm\ non\text{-}canc}$ (mg/kg-day)**		**$ADD_{derm\ canc}$ (mg/kg-day)**	
	adult	child	adult	child	adult	child	adult	child	adult	child	adult	child
Al	1.28×10^{-6}	2.98×10^{-6}	4.39×10^{-7}	2.56×10^{-7}	8.70×10^{-3}	8.12×10^{-2}	2.98×10^{-3}	6.96×10^{-3}	3.67×10^{-2}	1.93×10^{-1}	1.26×10^{-2}	1.65×10^{-2}
As	2.95×10^{-9}	6.89×10^{-9}	1.01×10^{-9}	5.90×10^{-10}	1.19×10^{-5}	1.05×10^{-4}	4.07×10^{-6}	9.03×10^{-6}	2.54×10^{-6}	1.33×10^{-5}	8.72×10^{-7}	1.14×10^{-6}
Ba	2.25×10^{-8}	5.24×10^{-8}	7.70×10^{-9}	4.49×10^{-9}	1.48×10^{-4}	1.26×10^{-3}	5.09×10^{-5}	1.08×10^{-4}	6.45×10^{-4}	3.38×10^{-3}	2.21×10^{-4}	2.90×10^{-4}
Be	1.09×10^{-10}	2.54×10^{-10}	3.73×10^{-11}	2.18×10^{-11}	7.09×10^{-7}	5.75×10^{-6}	2.43×10^{-7}	4.93×10^{-7}	3.12×10^{-6}	1.64×10^{-5}	1.07×10^{-6}	1.40×10^{-6}
Cd	2.32×10^{-10}	5.41×10^{-10}	7.94×10^{-11}	4.63×10^{-11}	1.49×10^{-6}	1.16×10^{-5}	5.11×10^{-7}	9.95×10^{-7}	6.65×10^{-9}	3.49×10^{-8}	2.28×10^{-9}	2.99×10^{-9}
Co	4.73×10^{-10}	1.10×10^{-9}	1.62×10^{-10}	9.47×10^{-11}	3.00×10^{-6}	2.25×10^{-5}	1.03×10^{-6}	1.93×10^{-6}	1.36×10^{-5}	7.13×10^{-5}	4.66×10^{-6}	6.11×10^{-6}
Cr(III)	1.81×10^{-8}	4.23×10^{-8}	6.22×10^{-9}	3.63×10^{-9}	1.14×10^{-4}	8.22×10^{-4}	3.89×10^{-5}	7.05×10^{-5}	5.21×10^{-4}	2.73×10^{-3}	1.78×10^{-4}	2.34×10^{-4}
Cr(VI)	1.81×10^{-8}	4.23×10^{-8}	6.22×10^{-9}	3.63×10^{-9}	1.12×10^{-4}	7.85×10^{-4}	3.84×10^{-5}	6.72×10^{-5}	5.21×10^{-4}	2.73×10^{-3}	1.78×10^{-4}	2.34×10^{-4}
Cu	2.51×10^{-8}	5.85×10^{-8}	8.60×10^{-9}	5.02×10^{-9}	1.53×10^{-4}	1.04×10^{-3}	5.25×10^{-5}	8.90×10^{-5}	7.20×10^{-4}	3.78×10^{-3}	2.47×10^{-4}	3.24×10^{-4}
Fe	8.15×10^{-6}	1.90×10^{-5}	2.79×10^{-6}	1.63×10^{-6}	4.91×10^{-2}	3.23×10^{-1}	1.68×10^{-2}	2.77×10^{-2}	2.34×10^{-1}	1.23×10^{0}	8.02×10^{-2}	1.05×10^{-1}
Li	4.25×10^{-9}	9.92×10^{-9}	1.46×10^{-9}	8.50×10^{-10}	2.53×10^{-5}	1.62×10^{-4}	8.67×10^{-6}	1.39×10^{-5}	1.22×10^{-4}	6.40×10^{-4}	4.18×10^{-5}	5.49×10^{-5}
Mn	1.26×10^{-7}	2.93×10^{-7}	4.31×10^{-8}	2.51×10^{-8}	7.38×10^{-4}	4.60×10^{-3}	2.53×10^{-4}	3.94×10^{-4}	3.61×10^{-3}	1.89×10^{-2}	1.24×10^{-3}	1.62×10^{-3}
Ni	4.93×10^{-9}	1.15×10^{-8}	1.69×10^{-9}	9.85×10^{-10}	2.86×10^{-5}	1.74×10^{-4}	9.80×10^{-6}	1.49×10^{-5}	1.41×10^{-6}	7.42×10^{-6}	4.85×10^{-7}	6.36×10^{-7}
Pb	1.72×10^{-8}	4.02×10^{-8}	5.91×10^{-9}	3.44×10^{-9}	9.88×10^{-5}	5.86×10^{-4}	3.39×10^{-5}	5.02×10^{-5}	4.95×10^{-4}	2.59×10^{-3}	1.70×10^{-4}	2.22×10^{-4}
Sn	2.52×10^{-9}	5.88×10^{-9}	8.63×10^{-10}	5.04×10^{-10}	1.43×10^{-5}	8.27×10^{-5}	4.89×10^{-6}	7.09×10^{-6}	7.23×10^{-5}	3.79×10^{-4}	2.48×10^{-5}	3.25×10^{-5}
Sr	2.83×10^{-8}	6.60×10^{-8}	9.70×10^{-9}	5.66×10^{-9}	1.59×10^{-4}	8.98×10^{-4}	5.43×10^{-5}	7.70×10^{-5}	8.13×10^{-4}	4.26×10^{-3}	2.79×10^{-4}	3.65×10^{-4}
V	6.04×10^{-9}	1.41×10^{-8}	2.07×10^{-9}	1.21×10^{-9}	3.35×10^{-5}	1.86×10^{-4}	1.15×10^{-5}	1.59×10^{-5}	1.74×10^{-4}	9.10×10^{-4}	5.95×10^{-5}	7.80×10^{-5}
Zn	1.17×10^{-6}	2.74×10^{-6}	4.02×10^{-7}	2.34×10^{-7}	6.41×10^{-3}	3.49×10^{-2}	2.20×10^{-3}	2.99×10^{-3}	3.37×10^{-2}	1.77×10^{-1}	1.15×10^{-2}	1.51×10^{-2}
Zr	9.17×10^{-10}	2.14×10^{-9}	3.14×10^{-10}	1.83×10^{-10}	4.96×10^{-6}	2.64×10^{-5}	1.70×10^{-6}	2.27×10^{-6}	2.63×10^{-5}	1.38×10^{-4}	9.02×10^{-6}	1.18×10^{-5}

– not applicable to the available data

References

1. Ośródka, L.; Godłowska, J.; Hajto, M.; Rozwoda, W.; Wojtylak, M. Determining the anemological conditions for the area of Kraków on the basis of data from the Institute of Meteorology and Water Management observation and measurement network (in Polish). Krakowski Holding Komunalny S.A. 2010. http://archiwum.dialogspoleczny.krakow.pl/files/objects/2061/35/Za%C4%B9%E2%80%9A%C3%84%E2%80%A6cznik%20nr%201%20-%20Analiza%20anemologiczna_09.02%20site.pdf. (Accessed on 21 July 2020).
2. *Change in the Study of Conditions and Directions for Spatial Development of the City of Krakow*. Volume 1: Determinants; Urząd Miasta Krakowa: Kraków, Poland, 2013. https://www.bip.krakow.pl/?dok_id=62484. (Accessed on 21 July 2020).
3. Regional Environmental Protection Inspectorate in Kraków, Poland. Wojewódzki Inspektorat Ochrony Środowiska w Krakowie. Available online: http://Kraków.pios.gov.pl/ (Accessed on 26 May 2020).
4. Gruszecka-Kosowska, A. Deposited particulate matter enrichment in heavy metals and related health risk: A case study of Kraków, Poland. *Proceedings*, **2020**, *44(1)*, 1–7. doi: 10.3390/IECEHS-2-06373.
5. Müller, G. Index of geoaccumulation in sediments of the Rhine River, *GeoJournal* **1969**, *2(3)*, 108–118.
6. Loska, K.; Wiechuła, D.; Korus, I. Metal contamination of farming soils affected by industry. *Environ Int* **2004**, *30*, 159–165.
7. Sutherland, R.A. (2000). Bed sediment-associated trace metals in an urban stream, Oahu, Hawaii. *Environ Geol* **2000**, *39(6)*, 611–627.
8. Ho, H.H.; Swennen, R.; Van Damme, A. Distribution and contamination status of heavy metals in estuarine sediments near Cua Ong Harbo, Ha Long Bay, Vietnam. *Geol Belg* **2010**, *13(1-2)*, 37–47.
9. Gong, Q.; Deng, J. Calculating pollution indices by heavy metals in ecological geochemistry assessment and as case study in parks of Beijing. *J China Univ Geosci* **2008**, *19(3)*, 230–241.
10. Chai, L.; Li, H.; Yang, Z.; Min, X.; Liao, Q.; Liu, Y.; Men, S.; Yan, Y.; Xu, J. Heavy metals and metalloids in the surface sediments of the Xiangjiang River, Hunan, China: distribution, contamination, and ecological risk assessment. *Environ Sci Pollut Res* **2017**, *24*, 874–885.
11. Liu, Y., Wang, Q., Zhuang, W., Yuan, Y., Jiao, K., Wang, M., & Chen, Q. (2018). Calculation of thallium's toxicity coefficient in the evaluation of potential ecological risk index: a case study. *Chemosphere* **2018**, *194*, 562–569.
12. Wang, N., Wang, A., Kong, L., & He, M. (2018). Calculation and application of Sb toxicity coefficient for potential ecological risk assessment. *Sci Total Environ* **2018**, *610–611*, 167–174.
13. Benson, N.U.; Adedapo, A.E.; Omowunmi, H.F.A.; Williams, A.B.; Udosen, E.D.; Ayejuyo, O.O.; Olajire, A.A. New ecological risk indices for evaluating heavy metals contamination in aquatic sediment: A case study of the Gulf of Guinea. *Regional Studies in Marine Science* **2018**, *18*, 44–56.
14. MacDonald, D.D.; Ingersoll, C.G.; Berger, T.A. Development and evaluation of consensus-based sediment quality guidelines for freshwater ecosystems. *Arch Environ Con Tox* **2000**, *39*, 20–31.
15. Kabata-Pendias, A. *Trace elements in soils and plants (4th ed.)*. Boca Raton: CRC Press, Taylor &Francis Group, 2011.
16. Håkanson, L. An ecological risk index for aquatic pollution control. A sedimentological approach. *Water Res* **1980**, *14*, 975–1001.
17. Rudnick, R.; Gao, S. Composition of the Continental Crust. In *Treatise on Geochemistry 2nd Edition*; Elsevier Science: Oxford, UK; San Diego, CA, USA: Elsevier Pergamon, **2014**.
18. *Exposure Factors Handbook: 2011 Edition*. National Center for Environmental Assessment, Washington, DC; US Environmental Protection Agency, 2011, EPA/600/R-09/052F.
19. *Supplemental guidance for developing soil screening levels for superfund sites*. Office of Emergency and Remedial Response. US Environmental Protection Agency, 2002, Washington, DC, USA, OSWER 9355, 4–24.
20. *Child-specific Exposure Factors Handbook*. National Center for Environmental Assessment Office of Research and Development. Washington, DC; US Environmental Protection Agency, 2008, EPA/600/R-06/096F.
21. *Risk assessment guidance for superfund, Vol. 3: Part A, Process for conducting probabilistic risk assessment*. Office of Emergency and Remedial Response, US Environmental Protection Agency, 2001, Washington, DC, USA.
22. Wcisło, E.; Bronder, J.; Bubak, A.; Rodríguez-Valdés, E.; Gallego, J.L.R. Human health risk assessment in restoring safe and productive use of abandoned contaminated sites. *Environ Int* **2016**, *94*, 436–448.
23. *Human Health Evaluation Manual, Supplemental Guidance: Update of Standard Default Exposure Factors.* Office of Solid Waste and Emergency Response, US Environmental Protection Agency, 2014, Washington, DC.

24. *Risk Assessment Guidance for Superfund Volume I: Human Health Evaluation Manual (Part F, Supplemental Guidance for Inhalation Risk Assessment)*, Final. Office of Superfund Remediation and Technology Innovation, OSWER Directive 9285,7-82, EPA-540-R-070-002, US Environmental Protection Agency, 2009, Washington, DC, USA.
25. *Regional Screening Level (RSL) Summary Table (TR=1E-06, HQ=1)* May 2020. US Environmental Protection Agency 2020, Washington DC, USA.
26. de Oliveira, B.F.A.; Ignotti, E.; Artaxo, P.; do Nascimento Saldiva, P.H.; Junger, W.L.; Hacon, S. Risk assessment of PM2.5 to child residents in Brazilian Amazon region with biofuel production. *Environ Health* **2012**, *11*:64.
27. Taiwo, A.M.; Awomeso, J.A.; Taiwo, O.T. Oremodu, B.D.; Akintunde, O.O.; Ojo, N.O.; Elegbede, O.O.; Olanrewaju, H.H.; Arowolo, T.A. Assessment of health risks associated with road dusts in major traffic hotspots in Abeokuta metropolis, Ogun state, southwestern Nigeria. *Stoch Environ Res Risk Assess* **2017**, *31(2),* 431–447.
28. Office of environmental health hazard assessment assessments. California Environmental Protection Agency, Office of Environmental Health Hazard Assessment, Oakland, CA., 2016. http://oehha.ca.gov/chemicals.
29. Regulation of the Minister of the Environment concerning the levels of certain substances in the air of 24 August 2012 (*OJ* 2012, Item 1031, in Polish). http://isap.sejm.gov.pl/isap.nsf/DocDetails.xsp?id=WDU20120001031.
30. Goddard, S.L.; Williams, K.R.; Robins, C.; Butterfield, D.M.; Brown, R.J.C. Concentration trends of metals in ambient air in the UK: a review. *Environ Monit Assess* **2019**, *191*, 683.
31. Air quality in Europe — 2019 report. *EEA Report No 10/2019*; Publications Office of the European Union, Luxembourg 2019.

© 2020 by the authors. Submitted for possible open access publication under the terms and conditions of the Creative Commons Attribution (CC BY) license (http://creativecommons.org/licenses/by/4.0/).
